# Rosamine derivatives of *o*-aminothiophenol-triacetate (S-APTRA): a new class of selective fluorescent sensors for Zn^2+^

**DOI:** 10.1039/d6ob00551a

**Published:** 2026-07-07

**Authors:** Laura L. Duncan, Dominic J. Black, Robert Pal, J. A. Gareth Williams

**Affiliations:** a Department of Chemistry, Durham University South Road Durham DH1 3LE UK laura.l.duncan@durham.ac.uk j.a.g.williams@durham.ac.uk

## Abstract

The Zn^2+^ ion has crucial roles in biology, such that the development of fluorescent probes for real-time monitoring of fluctuations in its concentration remains important. We describe a new class of probe that utilizes *ortho*-aminothiophenol-*N*,*N*,*S*-triacetate (S-APTRA) as the binding site for the metal ion, recently reported to bind Zn^2+^ with high selectivity over Ca^2+^ and Mg^2+^. The S-APTRA unit has been appended with a rosamine fluorophore by a sequence of formylation, condensation with 3-(dimethylamino)phenol, and oxidation. The resulting conjugate S-APTRA-Rosamine fluoresces only weakly in aqueous solution, but its emission is greatly enhanced by Zn^2+^, probably due to the suppression of a photoinduced electron transfer (PET) quenching process. The probe binds Zn^2+^ with a dissociation constant, *K*_d_, of 55.7 ± 1.2 nM, matching well with [Zn^2+^] in many biological cells, with very high selectivity over Ca^2+^ and Mg^2+^, and with attractively low-energy emission in the orange-red region. A proof-of-concept imaging experiment in NIH 3T3 cells reveals that the probe can successfully signal changes in [Zn^2+^] by confocal fluorescence microscopy. Meanwhile, a tetradentate analogue omitting the S-bonded carboxylate also responds to Zn^2+^ but the affinity is tempered by a factor of around 10^3^. Sulfoxide derivatives of the two systems show no response.

## Abstract 2

The linkage of a thiophenol-based azacarboxylate ligand to rosamine generates a new class of fluorescent sensors for Zn^2+^. Changes in [Zn^2+^] in live cells are successfully detected by fluorescence microscopy.

## Graphical abstract

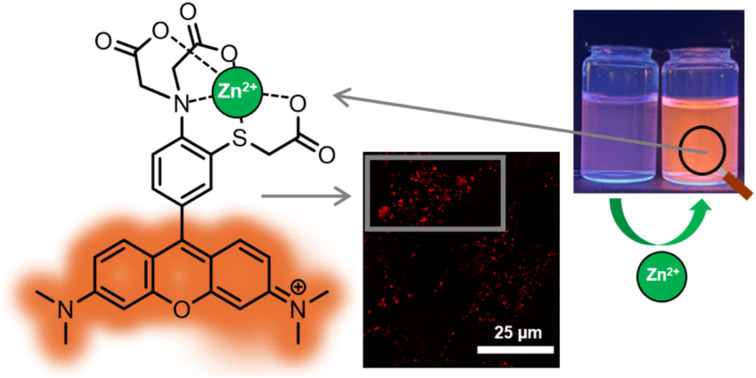

## Introduction

Zinc is an essential metal in living organisms and a key trace element in the human diet.^1^ Zinc has long been implicated with wound healing, fertility, growth, and the immune system, while its possible involvement in neurological conditions such as Parkinson's and Alzheimer's disease has more recently come under scrutiny.^2^ The Zn^2+^ ion has diverse cellular roles, the most well understood of which fall into the categories of structural or catalytic. For instance, the conformations of hundreds of proteins required for activity are maintained by the binding of amino acids (often histidine and cysteine) to Zn^2+^ in a pseudo-tetrahedral geometry.^3,4^ Meanwhile, a multitude of enzymes rely on the Lewis acidity associated with Zn^2+^. Several, like carbonic anhydrase (which catalyses the interconversion of CO_2_ and HCO_3_^−^), are crucial and found across all life forms.^5–7^ In these instances, the well-defined environment of the metal ion has typically been established with the aid of techniques such as protein crystallography and electron microscopy. There is much less known about the regulatory role of Zn^2+^ in cellular processes. The concentration of “free” Zn^2+^ (*i.e.*, the kinetically accessible, exchangeable pool of intracellular Zn^2+^) is typically very low in most cells, usually pM to nM, though concentrations in the μM range have been found in some tissues, particularly in the brain and pancreas.^8^ An increasing body of research has shown how the release of chelated Zn^2+^ may indeed be implicated in important cell signalling pathways.^9–12^

**Fig. 1:**
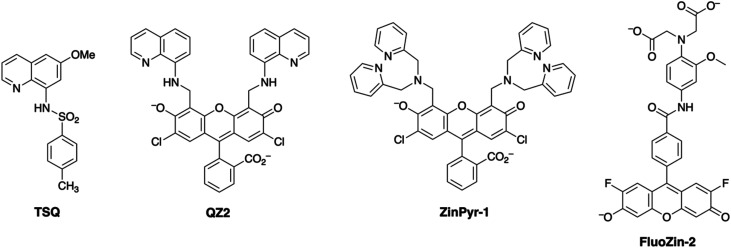
Representative fluorescent probes for Zn^2+^: TSQ is based on a quinoline sulfonamide; QZ2, ZinPyr-1, and FluoZin-2 feature metal-binding units based on 8-aminoquinoline, dipicolylamine, and *o*-aminophenol-*N*,*N*-diacetate chelating units, respectively, appended with fluorescein-based reporter groups to signal the binding through changes in fluorescence.

The widespread use of fluorescence microscopy in cell biology and physiology is greatly enriched by the development of fluorescent probes that respond to target analytes through the modulation of the fluorescence upon binding.^13–19^ Several such fluorescent sensors for Zn^2+^ have been successfully developed over the past 30 years.^20^ Simple 8-aminoquinoline derivatives^21^ such as TSQ {*N*-(6-methoxy-8-quinolyl)-*p*-toluenesulfonamide, Fig. 1} were adopted during the 1990s. Subsequently, the linkage of 8-aminoquinoline units to a fluorescein core to generate probes with affinities for Zn^2+^ in the μM range was achieved by Lippard and coworkers^22^ (*e.g.*, QZ2, Fig. 1; note that when discussing affinities in this paper, we adopt the convention of referring to dissociation constants, *K*_d_ ^23^). Meanwhile, related probes featuring the zinc-selective dipicolylamine (DPA) group, –N(CH_2_py)_2_, were developed that bind Zn^2+^ more strongly in the μM to nM range (*e.g.*, ZinPyr-1, Fig. 1).^24,25^ They continue to be widely adopted.^26–28^ There are also some systems that are based on azacarboxylate ligands (*e.g.*, FluoZin-2 ^29^). They are attractive particularly from the point of view that carboxylate-based probes can be loaded into cells as their acetoxymethyl (AM) esters,^30–32^ which are subsequently hydrolysed intracellularly leading to improved cellular retention and thus to enhanced contrast in imaging; on the other hand, they are more likely to suffer interference from Ca^2+^ and Mg^2+^. Nevertheless, all these probes feature binding units of relatively low denticity (usually bi-, tri- or tetradentate for the three classes shown), leaving space around the Zn^2+^ ion for endogenous biological molecules to also coordinate (including through chelation). The formation of such “ternary complexes” may hamper the accurate detection of “free” Zn^2+^.^33–35^

**Fig. 2:**
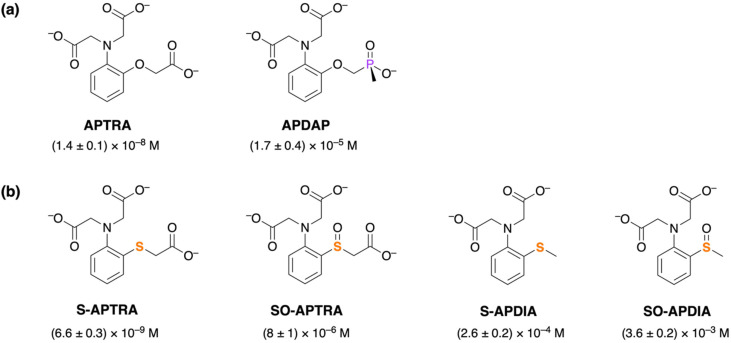
(a) The structures of *o*-aminophenol-*N*,*N*,*O*-triacetate, APTRA, and its phosphinate analogue, *o*-aminophenol-*N*,*N*-diacetate-*O*-methylene-methylphosphinate, APDAP. (b) The four sulfur-containing relatives of APTRA described in our earlier work,^36^ fluorescent derivatives of which have been targeted in the present study. The respective *K*_d_ values for binding of Zn^2+^ are shown underneath each compound.^36,39,43^

Given the above limitations, it is evident that it would be desirable to develop new, carboxylate-based, Zn^2+^-specific binding units that are at least pentadentate. With these aspirations in mind, we recently described the new ligand *o*-aminothiophenol-*N*,*N*,*S*-triacetate, which we refer to as S-APTRA (Fig. 2).^36^ It is an analogue of the pentadentate azacarboxylate ligand *o*-aminophenol-*N*,*N*,*O*-triacetate (APTRA) that has been quite widely adopted for targeting Mg^2+^, despite binding Ca^2+^ and Zn^2+^ significantly more strongly.^37–41^ The substitution of the phenolic oxygen atom by sulfur favours the binding of the softer Zn^2+^ ion, rendering S-APTRA highly selective for Zn^2+^ over Mg^2+^ and Ca^2+^. S-APTRA fulfils the requirement of pentadenticity to inhibit ternary complex formation, whilst the negatively charged carboxylates promote aqueous solubility and are amenable to protection as AM esters. Moreover, the nM binding affinity for Zn^2+^ could be attenuated (and the selectivity modulated) either by oxidation of the sulfur to a sulfoxide (to give SO-APTRA with *K*_d_ for Zn^2+^ in the µM range), or omission of one of the carboxylates (S-APDIA, µM range). Meanwhile, the combination of both changes (giving SO-APDIA) further suppressed the binding (*K*_d_ in the mM range).

In the present work, we set out to link the S-APTRA unit and its derivatives to a biocompatible, visibly absorbing and emitting, and aqueous-compatible fluorophore, with a view to exploring the utility of the resulting “receptor–fluorophore” compounds as fluorescent sensors for Zn^2+^ in aqueous media.

## Results and discussion

**Scheme 1:**
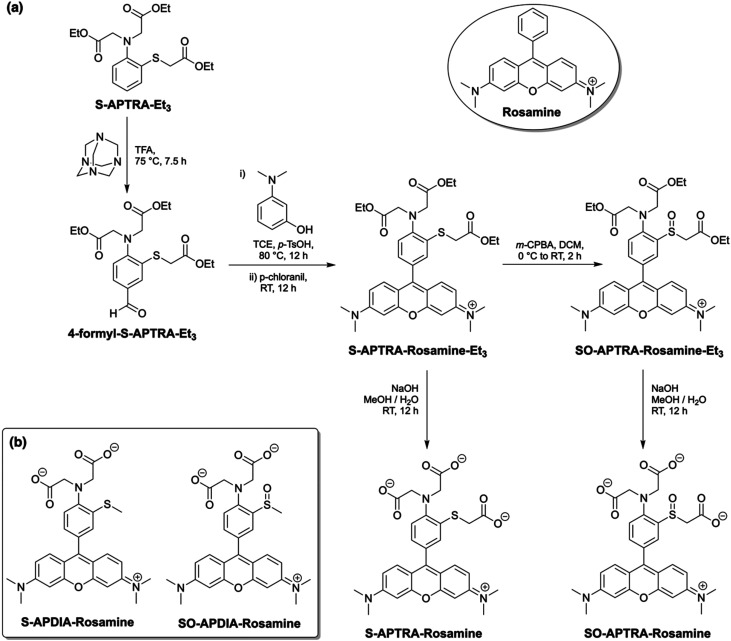
(a) Synthesis of S-APTRA-Rosamine and SO-APTRA-Rosamine, starting from the previously reported compound S-APTRA-Et_3_. (b) Structures of the corresponding compounds omitting the S-bonded carboxylate, S-APDIA-Rosamine and SO-APDIA-Rosamine, which were prepared similarly from S-APDIA-Et_2_ (Fig. 2b). Rosamine itself is shown circled at the top.

We chose rosamine (circled structure in Scheme 1) as a target fluorophore for attachment to the S-APTRA binding unit. Rosamines and rhodamines are very widely used diaminoxanthene-based fluorophores that absorb light strongly and emit efficiently in the 500–700 nm region. These relatively long wavelengths (*e.g.*, compared to their fluorescein cousins) are attractive for use in biological media and applications such as bioimaging, as light in this region of the spectrum has deeper tissue penetration than shorter wavelengths, being both scattered to a lesser extent (∝*λ*^−4^) and less likely to be absorbed by endogenous molecules. Rosamines lack the carboxylate unit of the pendent phenyl ring of rhodamines, and so they do not suffer from the competitive lactonization to a non-fluorescent tautomer that is sometimes problematic for rhodamines under acidic conditions. Rosamine has been used to prepare fluorescent probes for metal ions, including recent work by Hiruta and co-workers in which it was attached to *o*-aminophenol-*N*,*N*-diacetate-*O*-methylene-methylphosphinate (APDAP, Fig. 2), a phosphinate analogue of APTRA.^42,43^

The classical synthesis of rosamines is through the condensation of 3-aminophenols with a benzaldehyde in a 2 : 1 molar ratio. Although limited in substrate scope and accompanied by poor yields, the methodology appeared to be appropriate for the target compound, provided that an aldehyde-functionalized derivative of S-APTRA could be accessed. We initially attempted to formylate the tris ethyl ester of S-APTRA (S-APTRA-Et_3_ ^36^) by a Vilsmeier-Haack reaction with POCl_3_ in DMF. However, conversion was limited to around 50%, and the remarkably similar *R*_f_ values of the product and starting material on silica limited the achievable yields after chromatographic purification to <10%. In contrast, essentially quantitative conversion to the aldehyde was achieved by a Duff reaction^44^ with hexamethylenetetramine in TFA and subsequent hydrolysis of the iminium ion. The condensation of 4-formyl-S-APTRA-Et_3_ with 3-(dimethylamino)phenol (2.5 equiv.) in the presence of *p*-toluenesulfonic acid, followed by oxidation of the resulting intermediate with *p*-chloranil, gave S-APTRA-Rosamine-Et_3_ in a low but adequate yield (Scheme 1a). The target ligand S-APTRA-Rosamine was obtained as an intensely purple solid by base hydrolysis of the tris ethyl ester in aqueous methanol.

The analogue omitting the S-bonded carboxylate, namely S-APDIA-Rosamine (Scheme 1b), was obtained in the same way from S-APDIA-Et_2_. The sulfoxide derivatives SO-APTRA-Rosamine and SO-APDIA-Rosamine were prepared by oxidation of S-APTRA-Rosamine-Et_3_ and S-APDIA-Rosamine-Et_3_, respectively, with *meta*-choroperbenzoic acid (*m*-CPBA), followed by base-catalysed ester hydrolysis (Scheme 1).

### Absorption and emission spectra

**Fig. 3:**
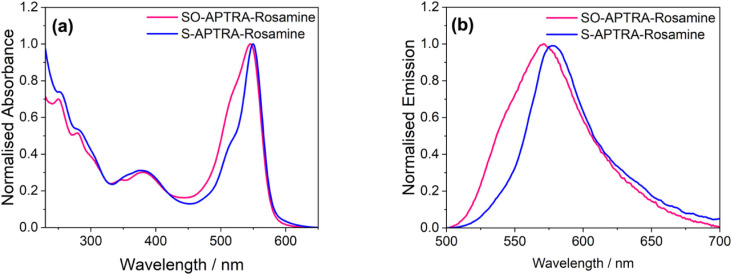
(a) Absorption spectra of S-APTRA-Rosamine (blue) and SO-APTRA-Rosamine (pink) at 295 K in buffered aqueous solution (50 mM HEPES, pH 7.2, 100 mM KCl). (b) Corresponding emission spectra under the same conditions, *λ*_ex_ = 480 nm.

All spectra were recorded at 295 K in buffered aqueous solution (50 mM HEPES, pH 7.2, in the presence of 100 mM KCl), to partially mimic a cellular or, at least, a biologically relevant environment. The absorption spectrum of S-APTRA-Rosamine closely resembles that of rosamine itself,^45^ being dominated by the lowest-energy, intense band centred at 550 nm (Fig. 3). An aromatic vibrational shoulder to high-energy is visible, typical of such chromophores.

The emission spectrum displays one band centred at 580 nm, with a hint of the vibrational component to lower energy (Fig. 3a). The reported emission maximum of rosamine itself is 570 nm, but that value applies to ethanol rather than water.^45^ The small shift may simply reflect the differing solvation of the S_1_ state in more polar media, or it could be due to a small influence of the substituents on the pendent phenyl ring. That these substituents do have at least some effect on the excited state properties is apparent from the corresponding spectra of the sulfoxide analogue SO-APTRA-Rosamine (Fig. 3b). Its absorption maximum is marginally blue-shifted relative to S-APTRA-Rosamine, but more strikingly, the relative intensity of the vibrational shoulder is increased. The emission maximum is likewise marginally blue-shifted and the band is broader. Oxidation of the sulfur may slightly increase the energy of the rosamine-localized excited state by suppressing electron donation from the pendent phenyl ring. The emission of both compounds is weak, with fluorescence quantum yields, *Φ*_f_, of 3 and 5% respectively. The suppressed emission compared to rosamine (*Φ*_f_ = 87% in EtOH^45^) indicates that the substituents on the pendent phenyl ring quench the excited state. A photoinduced electron transfer (PET) quenching mechanism is anticipated from the aniline nitrogen, as observed for innumerable amine-appended fluorophores. The higher *Φ*_f_ of the sulfoxide compared to the sulfide is likely because, in the latter, the reducing power of the aniline (and hence its quenching power) will be enhanced by electron donation from the sulfur atom, but such an effect is lost upon oxidation at S. The differing effects of protonation and metal binding (discussed in the next sections) also support a contribution to the PET quenching mechanism from the sulfur. We note that the excitation spectra of the four ligands closely match their absorption spectra, confirming the origin of the emission from the single compound in solution each case (Fig. S1).

### Effect of protonation

**Fig. 4:**
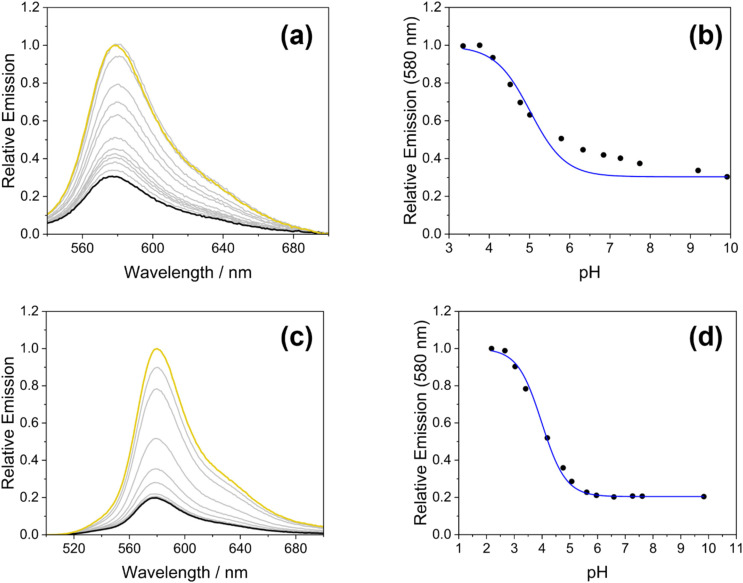
(a) The evolution of the emission spectrum of S-APTRA-Rosamine in aqueous solution (100 mM KCl), from basic (black line) to acidic (yellow line) conditions; *λ*_ex_ = 480 nm. Spectra at intermediate pH values are shown in grey. (b) The fitting of the emission intensity to pH, from which a p*K*_a_ of 5.0 was estimated. (c) and (d) Corresponding spectra and data for SO-APTRA-Rosamine, λ_ex_ = 500 nm, giving a p*K*_a_ of 4.0.

The addition of acid to the compounds in solution leads to no change in the absorption spectrum, nor to the emission *λ*_max_. The emission intensity is, however, significantly increased, by around 3-fold for S-APTRA-Rosamine and 5-fold for SO-APTRA-Rosamine (Fig. 4). The most basic site on the molecules is evidently the aniline nitrogen, the protonation of which will suppress the PET effect postulated above. On the other hand, thioethers are extremely weak bases and do not normally protonate under aqueous acidic conditions. Some remaining PET quenching might therefore be anticipated for S-APTRA-H^+^-Rosamine, but not for the corresponding sulfoxide. This may account for the lesser protonation-induced enhancement of the emission observed for the former. The fluorescence lifetime of 2.1 ns recorded for the two compounds was not changed by protonation, an observation that is consistent with the static quenching model typically found for PET systems.^46^

For S-APTRA-Rosamine, the fitting of the emission intensity against pH gave a rather shallow endpoint, but it sufficed for an estimate to be made of p*K*_a_ = 5.0 (Fig. 4a, inset). This value is similar to that of 5.2 found for S-APTRA by absorption spectroscopy,^36^ indicating that the presence of the rosamine unit has little effect on the electron density at the aniline nitrogen. The p*K*_a_ of SO-APTRA-Rosamine determined in the same way (Fig. 4b) was 4.0. Its lower basicity compared to the parent compound can be attributed to the loss of the electron-donating influence of the *ortho*-disposed sulfide and the introduction of the electron-withdrawing sulfoxide unit in its place. The p*K*_a_ values of both compounds are outside the usual physiological range, which is a key attribute for practicable metal ion probes to rule out interference from protonation.

### Effect of metal ions: Zn^2+^, Ca^2+^, and Mg^2+^

**Fig. 5:**
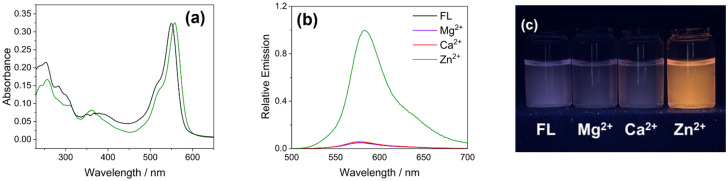
(a) Absorption spectrum of S-APTRA-Rosamine (10 μM) in the presence of Zn^2+^ (green line) compared to that with no Zn^2+^ (black line). (b) Emission spectra of S-APTRA-Rosamine (10 μM; *λ*_ex_ = 480 nm) in the presence of Zn^2+^ (1 nM ZnSO_4_; green line), or Ca^2+^ (10 μM CaCl_2_; red line), or Mg^2+^ (1 mM MgCl_2_; purple line) compared to the spectrum with no metal ions present (black line). Note that the latter three spectra are essentially identical to one another. All spectra were recorded in buffered aqueous solution (50 mM HEPES, pH 7.2, 100 mM KCl). (c) A photograph of the four solutions of (b) under long-wavelength UV irradiation.

The effect of Zn^2+^, Ca^2+^, and Mg^2+^ on the absorption and emission spectra of S-APTRA-Rosamine was investigated, initially at concentrations of 10^−9^, 10^−5^, and 10^−3^ M, respectively, corresponding to the upper end of their typical intracellular values (Fig. 5). At these concentrations, neither Ca^2+^ nor Mg^2+^ led to any detectable change, whereas Zn^2+^ induced a dramatic “turn-on” response, with the intensity being increased by 20-fold (Fig. 5b and c). The emission *λ*_max_ is essentially unchanged, but a small redshift is observed in the absorption spectrum, again for Zn^2+^ only (Fig. 5a and Fig. S2). The increase in the emission intensity is consistent with the binding of the Zn^2+^ ion to the S-APTRA nitrogen atom and the resulting suppression the PET quenching pathway. However, the enhancement factor is superior to that observed upon protonation (around 3-fold, Fig. 4a), supporting the notion of an additional contribution to PET quenching from the sulfur lone pair. Its contribution will be inhibited upon coordination to Zn^2+^, through the expected binding of the sulfur atom of the pentadentate ligand to the metal ion, whereas protonation is confined to the nitrogen atom.

**Fig. 6:**
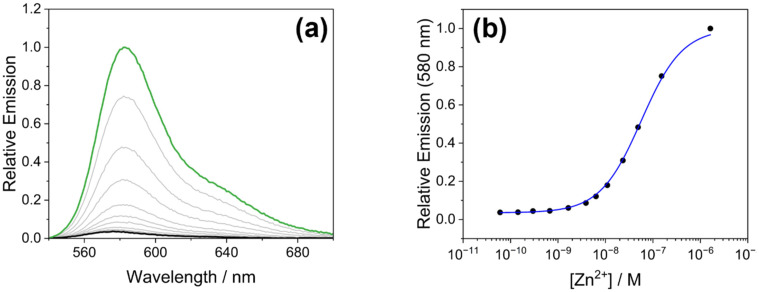
(a) Fluorescence spectra of a representative titration of S-APTRA-Rosamine (10 μM) with ZnSO_4_, λ_ex_ = 520 nm, and (b) the associated fitting of experimental data points to a 1 : 1 binding model, in buffered aqueous solution (50 mM HEPES, pH 7.2, 100 mM KCl) in the presence of EGTA (1 mM). The black line in (a) is the starting spectrum with no metal present and the green line that at the end; the spectra at intermediate concentrations of Zn^2+^ are in grey.

The binding affinities were quantified through fluorescence titrations, in which the emission intensity at 580 nm was monitored as a function of [M^2+^]. For Zn^2+^ (Fig. 6), the very strong binding necessitated the use of a competitive binding procedure, whereby the titration was carried out in the presence of a fixed concentration of EGTA^47^ (ethylene glycol bis(β-aminoethylether)-*N*,*N*,*N*',*N*'-tetracetate, as used previously for S-APTRA itself;^36^ details are given in the SI). Using this method, a *K*_d_ value of 55.7 ± 1.2 nM was determined, assuming a 1 : 1 binding stoichiometry. That assumption is supported by a Job plot (“method of continuous variation”, Fig. S3) and by analogy with conclusions for S-APTRA from ^1^H NMR titrations with Zn^2+^.^36^ The affinity of the rosamine derivative for Zn^2+^ is somewhat attenuated compared to the parent (for which *K*_d_ = 6.6 ± 0.3 nM under the same conditions). This may be due to the combined effects of modestly electron-withdrawing character of the fluorophore removing electron density from the APTRA unit,^40^ coupled with the positive charge on the fluorophore generating an element of electrostatic repulsion of the metal cation.

**Fig. 7:**
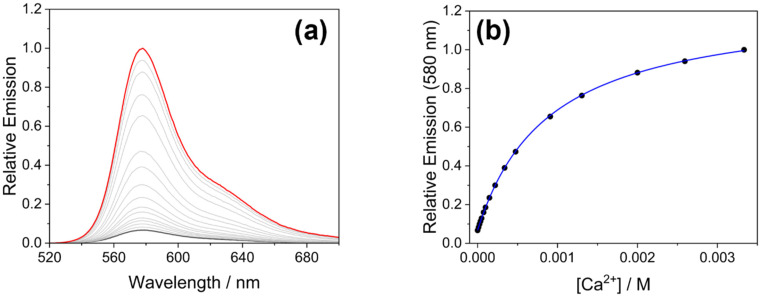
(a) Fluorescence spectra of a representative titration of S-APTRA-Rosamine (10 μM) with CaCl_2_, λ__ex_ = 500 nm_, and (b) the associated fitting of experimental data points to a 1 : 1 binding model, in buffered aqueous solution (50 mM HEPES, pH 7.2, 100 mM KCl). The black line in (a) is the starting spectrum with no metal present and the red line that at the end; the spectra at intermediate concentrations of Ca^2+^ are in grey.

Although neither Mg^2+^ nor Ca^2+^ led to any significant change in emission at their upper cellular concentrations, higher concentrations of Ca^2+^ did induce a “turn-on” emission response, from which a *K*_d_ of 879 ± 23 μM was determined (Fig. 7). The binding is thus weak, about 10^3^ times weaker than APTRA, and the *K*_d_ value far exceeds intracellular [Ca^2+^], such that the compound would not suffer from Ca^2+^ interference if used as a Zn^2+^ probe. The Ca^2+^ binding is again somewhat weaker than that of the parent S-APTRA (*K*_d_ = 326 ± 7 μM), no doubt for the same reasons as for Zn^2+^ above (electron-withdrawing nature and positive charge of the fluorophore). No *K*_d_ could be determined for Mg^2+^ owing to the minimal change in the spectrum even for large [Mg^2+^] (Fig. S4).

**Fig. 8:**
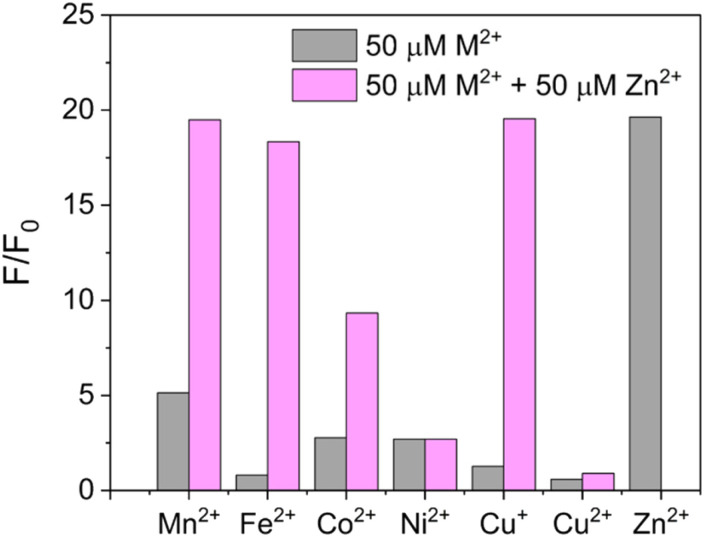
Metal selectivity plot showing the ratio of the emission intensity at 580 nm of S-APTRA-Rosamine (10 μM) with 50 µM of the indicated metal ions added, relative to that of the free ligand (grey bars), and the corresponding values after subsequent addition of 50 µM Zn^2+^ (pink); in buffered aqueous solution (50 mM HEPES, pH 7.2, 100 mM KCl). All ions were added as their chloride salts, except Zn^2+^ (ZnSO_4_) and Cu^+^ (from a 1 mM stock solution of [Cu(MeCN)_4_][PF_6_] in MeCN).

We also checked for interference from a selection of other 1^st^ row transition metal ions: Mn^2+^, Fe^2+^, Co^2+^, Ni^2+^, Cu^+^, and Cu^2+^ (Fig. 8 and Fig. S5). Upon addition of 50 µM of each of these metal ions, the emission intensity remained much lower than that observed in the presence of the same concentration of Zn^2+^. When Zn^2+^ (50 μM) was then added to the resulting solutions, the emission intensity completely recovered for Mn^2+^, Fe^2+^ and Cu^+^ indicating no interference from these three metal ions. Conversely, only partial recovery of fluorescence was observed for Co^2+^ and none for Ni^2+^ and Cu^2+^. These three cations are well-known fluorescence quenchers, and such an effect is typical for established fluorescent probes.^23,48,49^ Nevertheless, the free ion concentrations of these metal ions are very low in mammalian cells, such that any interference from them during the detection of Zn^2+^ using the probe would be expected to be minimal.

### Effect of reduced denticity and sulfur oxidation

**Fig. 9:**
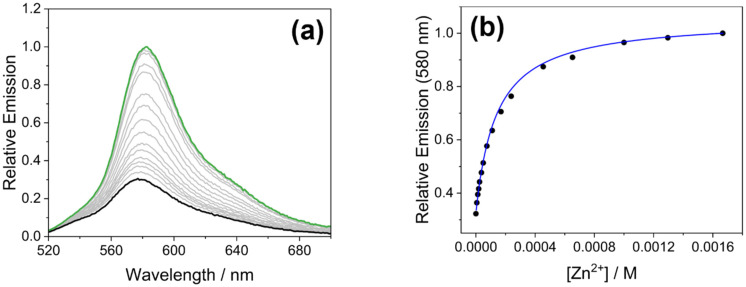
(a) Fluorescence spectra of a representative titration of S-APDIA-Rosamine (10 μM) with ZnSO_4_, λex = 500 nm, and (b) the associated fitting of experimental data points to a 1 : 1 binding model, in buffered aqueous solution (50 mM HEPES, pH 7.2, 100 mM KCl). The black line in (a) is the starting spectrum with no metal present and the green line that at the end; the spectra at intermediate concentrations of Zn^2+^ are in grey.

For the tetradentate derivative S-APDIA-Rosamine, the absorption and emission spectra are essentially identical to that of S-APTRA-Rosamine (Fig. S6). As expected, the truncation of the –CH_2_CO_2_^−^ sidearm of the sulfur atom to –CH_3_ does not significantly influence the electronics of the chromophore. As for S-APTRA-Rosamine, the addition of Zn^2+^ again leads to an enhancement in the emission intensity, but the binding is now over 1000× weaker (*K*_d_ = 130 ± 10 µM; Fig. 9 and S7). The decrease (also observed for S-APDIA by absorption spectroscopy) reflects the lower denticity reducing the contribution of the chelate effect to binding. The addition of Ca^2+^ or Mg^2+^ led to no detectable change in the emission or absorption spectra (Fig. S7 and S8).

For the sulfoxide derivative SO-APTRA-Rosamine, there is no significant change in the absorption or emission spectra upon the addition of Mg^2+^, Ca^2+^ or Zn^2+^ even at quite high concentrations (Fig. S9 and S10). In the case of SO-APTRA, the binding of Zn^2+^ had been found to be around 4 to 5 orders of magnitude weaker than for S-APTRA, but still detectable through the modulation of the UV-visible absorption spectrum.^36^ Presumably, for the rosamine derivative of the sulfoxide, the binding is weakened further and/or it does not significantly involve the nitrogen lone pair. Not surprisingly, the sulfoxide derivative SO-APDIA-Rosamine likewise showed no change in the emission, absorption or excitation spectra upon the addition of Mg^2+^, Ca^2+^ or Zn^2+^ (Fig. S11 and S12). The optical properties of the four ligands and their responses to Zn^2+^ are summarised in Table 1.

**Table 1:** Summary of the *K*_d_ values for the ligands with Zn^2+^ in aqueous solution at 295 K (50 mM HEPES, pH 7.2, 100 mM KCl)

	*K*_d_ Zn^2+^ a	Absorption^b^*λ*_max_/nm (*ε*/M^−1^ cm^−1^)	Emission *λ*_max_/nm
S-APTRA-Rosamine	55.7 ± 1.2 nM	255 (19 200), 284 (13 700), 383 (8080), 514 ^sh^ (11 900), 550 (26 100)	580
SO-APTRA-Rosamine	ND^c^	255 (15 600), 284 (11 600), 518 sh (16 800), 550 (22 100)	540^sh^, 577
S-APDIA-Rosamine	130 ± 10 μM	255 (21 700), 285 (14 700), 382 (5870), 516 ^sh^ (17 700), 550 (31 900)	580
SO-APDIA-Rosamine	ND^c^	256 (22 100), 283 (17 800), 348 (6900), 393 (7300), 522 ^sh^ (26 000), 551 (46 700)	540^sh^, 578

a Error values indicate the standard error of the mean for at least three separate titrations.

b Bands centred at *λ* > 240 nm are listed; sh = shoulder.

c *K*_d_ values were not determinable in these instances, due to there being almost no influence of Zn^2+^ on the emission spectrum.

### Practical application of S-APTRA-Rosamine in live cell imaging: proof of concept

**Fig. 10:**
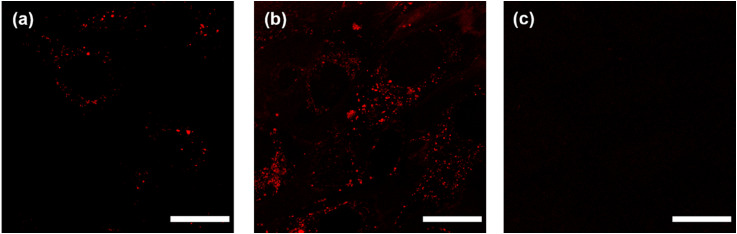
Representative live cell imaging of NIH 3T3 cells after incubation with S-APTRA-Rosamine at 2 μM for 2 h at 37 °C, (*λ*_ex_ = 543 nm, *λ*_em_ = 560–800 nm). (a) Initial image in the absence of added Zn^2+^. (b) Corresponding image after treatment with ZnSO_4_ (2 μM). (c) Image after subsequent treatment with TPEN (20 μM) for 30 min. Scale bar = 25 μm.

It is clear that the S-APTRA-Rosamine derivative displays the sought-after behaviour of strong binding of Zn^2+^ and high selectivity over Ca^2+^ and Mg^2+^. It responds through the modulation of fluorescence in an attractively low-energy region of the spectrum (tailing into the deep red), and with excitation possible at biocompatible long wavelengths >550 nm. Meanwhile, the Zn^2+^ affinity can be attenuated, if desired, using the tetradentate analogue S-APDIA-Rosamine. (Although its binding would be too weak in most biological applications, it could be suitable in some environmental settings with higher [Zn^2+^] under analysis). We therefore sought to establish whether S-APTRA-Rosamine could respond to Zn^2+^ ions in a mammalian cell environment, using fluorescence microscopy. Cells of the widely used NIH 3T3 line (mouse embryonic skin fibroblast cells) were incubated at 37 °C with S-APTRA-Rosamine dissolved in the growth medium at a concentration of 2 μM. The compound was determined to be non-toxic to the cells at this concentration, as illustrated by cytotoxicity assays (Fig. S13). The fluorescence of the probe in the cells could be readily detected through the microscope after incubation for 2 h (Fig. 10a). Treatment of the cells with ZnSO_4_ (2 μM) led to a clear-cut enhancement of the emission (Fig. 10b), implying that the probe binds to the newly introduced Zn^2+^ ions. As a further confirmation, the cells were then treated for 30 min with *N*,*N*,*N*′,*N*′-tetrakis(2-picolyl)ethylenediamine (TPEN), a widely used cell-permeable ligand with a high affinity for Zn^2+^, after which no fluorescence could be detected (Fig. 10c; corresponding transmission images for Fig. 10a–c are given in Fig. S14). This observation is consistent with the expected sequestration of Zn^2+^ by TPEN, which has a *K*_d_ in the sub-picomolar range and will therefore easily outcompete the probe.

**Fig. 11:**
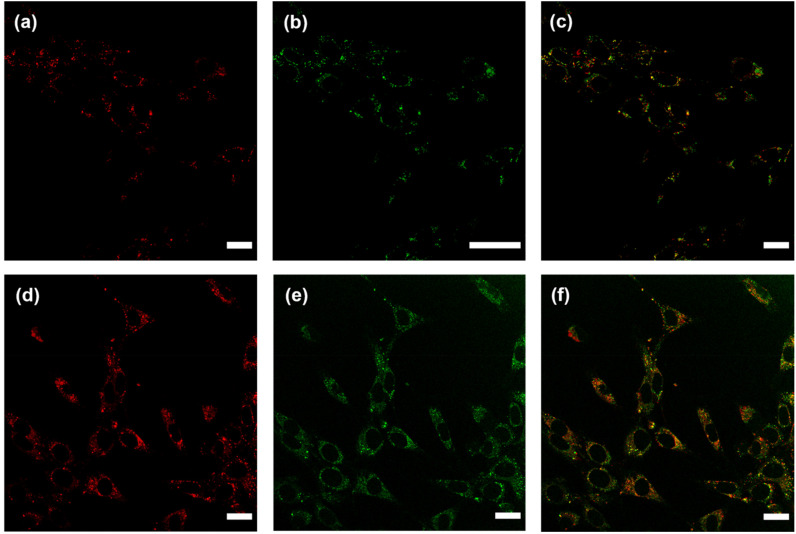
Co-stained fluorescence images of NIH 3T3 cells incubated with S-APTRA-Rosamine and co-stained with LysoTracker™ Green DND-26 and BioTracker™ 488 Green Lipid Droplet Dye: (a) S-APTRA-Rosamine (2 μM, 2 hours incubation, *λ*_ex_ = 543 nm, *λ*_em_ = 560–800 nm) (b) LysoTracker™ Green DND-26 (200 nM, 30 minutes incubation, *λ*_ex_ = 488 nm, *λ*_em_ = 500–520 nm). (c) RGB merge, PCC = 0.38. Scale bar = 25 μm. Separate set of measurements: (d) S-APTRA-Rosamine (2 mM, 2 hours incubation *λ*_ex_ = 543 nm, *λ*_em_ = 560–800 nm) (e) BioTracker™ 488 Green Lipid Droplet Dye (500 nM, 1 hour incubation *λ*_ex_ = 488 nm, *λ*_em_ = 500–520 nm). (f) RGB Merge PCC = 0.78. Scale bar = 25 μm.

The images suggest that S-APTRA-Rosamine (or at least its Zn^2+^ complex) accumulates primarily in lipid droplets. Co-localisation studies were conducted using a green-emitting lipid droplet stain and, separately, with LysoTracker Green (localises to the lysosomes) (Fig. 11, the corresponding transmission images are shown in Fig. S15). The Pearson correlation coefficients of 0.78 and 0.38 support predominant localisation to the lipid droplets rather than the lysosomes.

## Conclusion

The study shows that thiophenol-based analogues of APTRA can be readily derivatised with a xanthene-based fluorophore using a straightforward sequence of formylation followed by condensation. The rosamine conjugate S-APTRA-Rosamine, responds to Zn^2+^ in aqueous media through a large enhancement of the fluorescence intensity in the orange-red region of the spectrum when excited in the blue or green. The signalling mechanism likely involves the suppression of a PET quenching pathway when the metal ion interacts with the electron-rich sulfur and nitrogen atoms. The probe displays impressive selectivity over Ca^2+^ and Mg^2+^, the two most competitive divalent ions in typical cells. The tetradentate analogue S-APDIA-Rosamine, lacking the S-bonded acetate, also binds Zn^2+^ selectively, but less avidly, while oxidation to the corresponding sulfoxides further suppresses binding. As a result, the probes could potentially be used for the dual-sensing of Zn^2+^ and reactive oxygen species. The influence of the fluorescent reporter group on binding affinities is often rather overlooked in the design of metal ion probes. Here, the metal binding is weakened somewhat compared to the parent ligands, for all four systems. Nevertheless, for S-APTRA-Rosamine, the *K*_d_ of 55.7 ± 1.2 nM remains well suited for detection of changes in [Zn^2+^] at the concentrations that prevail in many cellular environments. The preliminary, proof-of-concept imaging experiments carried out here in NIH 3T3 cells confirms the applicability. Cell permeability could be expected to be improved for future studies through the preparation of AM esters, which is not possible for DPA-based probes, for instance.^50^ We anticipate that the systems will make an attractive addition to the toolbox of methods for monitoring Zn^2+^ in cell biology.

## Experimental section

General information about synthetic, spectroscopic, and imaging methods are given in the SI. The synthesis and characterisation of the rosamine derivatives and their immediate precursors are given below. Details for the hydrolysed compounds (*i.e.*, the carboxylate ligands), together with the procedures used to prepare known intermediates and the ^1^H and ^13^C NMR spectra of all new compounds, are provided in the SI.

### 4-Formyl-S-APTRA-Et_3_


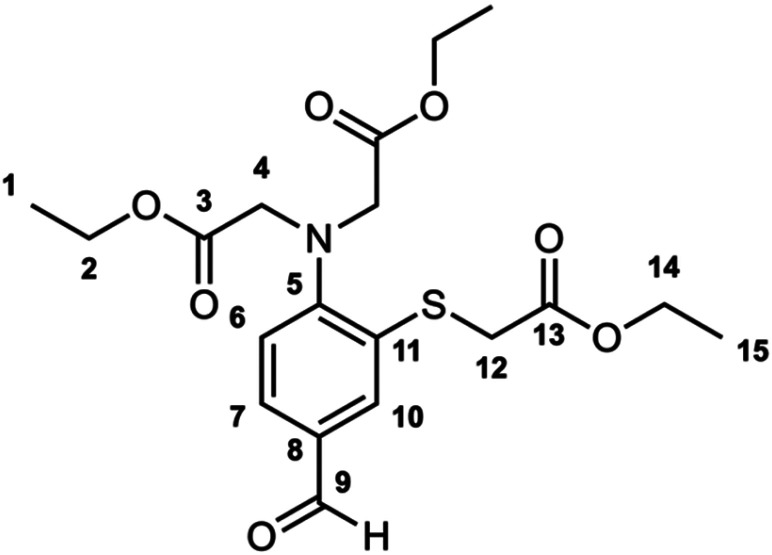
 Hexamethylenetetramine (0.16 g, 1.1 mmol) was added to a solution of S-APTRA-Et_3_ (0.15 g, 0.38 mmol) in TFA (2.0 cm^3^) at 0 °C under a nitrogen atmosphere. The mixture was refluxed for 7.5 h, then neutralised with saturated NaHCO_3_ (aq) and diluted with brine. The crude product was extracted into ethyl acetate (3 × 10 cm^3^) and washed with brine (10 cm^3^) before drying over MgSO_4_. The solvent was removed under reduced pressure, and the residue was deemed to be of sufficient purity for carrying forward to the next step without further purification. ^1^H (599 MHz, CDCl_3_): *δ*_H_ 9.85 (1H, s, H^9^), 7.89 (1H, d, *J* 1.9, H^10^), 7.68 (1H, dd, *J* 8.3, 1.9, H^7^), 7.19 (1H, d, *J* 8.3, H^6^), 4.27 (4H, s, H^4^), 4.16 (4H, q, *J* 7.2, H^2^), 4.10 (2H, q, *J* 7.2, H^14^), 3.73 (2H, s, H^12^), 1.24 (6H, t, *J* 7.2, H^1^), 1.18 (3H, t, *J* 7.2, H^15^). ^13^C (151 MHz, CDCl_3_): *δ*_C_ 190.5 (C^9^), 170.1 (C^3^), 169.3 (C^13^), 154.9 (C^5^), 134.7 (C^10^), 131.7 (C^8^), 128.7 (C^11^), 129.5 (C^7^), 121.9 (C^6^), 61.5 (C^14^), 61.1 (C^2^), 53.6 (C^4^), 35.1 (C^12^), 14.1 (C^1^), 14.0 (C^15^). ESI-LRMS *m*/*z* 412.23 ([C_19_H_25_NO_7_S + H]^+^, 100%). ESI-HRMS calculated for [C_19_H_26_NO_7_S]^+^ 412.1429, found 412.1430.

### S-APTRA-Rosamine-Et_3_


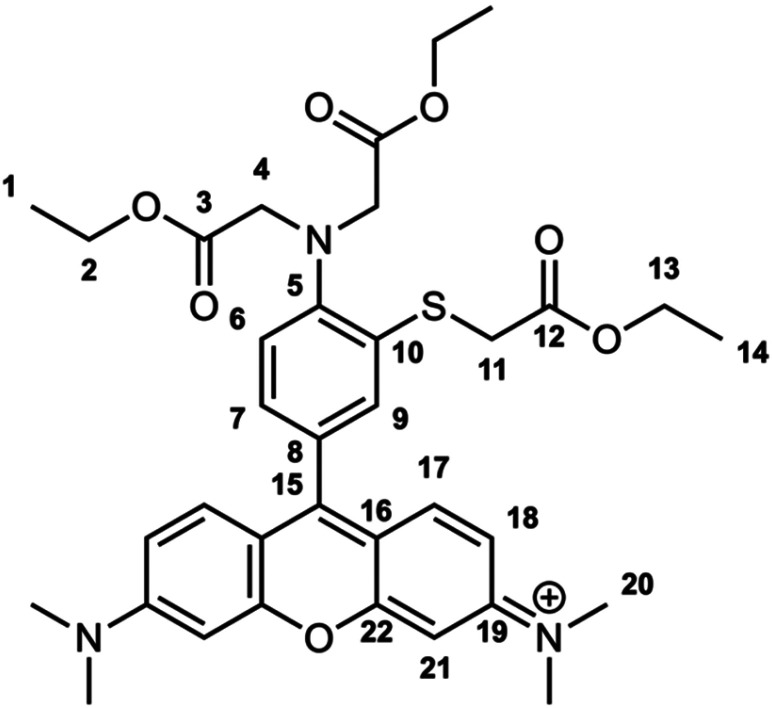
 4-Formyl-S-APTRA-Et_3_ (0.57 g, 1.3 mmol) and 3-(dimethylamino)phenol (0.44 g, 3.2 mmol) were combined in TCE (10 cm^3^) under nitrogen. *p*-Toluenesulfonic acid monohydrate (0.44 g, 2.3 mmol) was added, and the solution was heated at 80 °C for 24 h. After addition of *p*-chloranil (0.35 g, 1.4 mmol), the solution was stirred at RT for 12 h. The solvents were removed under reduced pressure with the resulting crude product diluted with DCM and washed with H_2_O (×2) and brine. After drying (MgSO_4_), the organic solvents were removed under reduced pressure to give a purple solid which was purified using column chromatography on silica (gradient from 100% DCM to 20% MeOH, 80% DCM) and then further using preparative HPLC (gradient from 30% MeCN, 70% H_2_O to 100% MeCN) (91 mg, 13%). ^1^H (599 MHz, CDCl_3_): *δ*_H_ 7.50–7.49 (1H, m, H^9^), 7.10–7.05 (1H, m, H^7^), 7.03–7.01 (1H, m, H^6^), 6.96–6.93 (2H, m, H^17^), 6.45–6.42 (2H, m, H^18^), 6.41–6.38 (2H, m, H^21^), 4.15–4.09 (8H, m, H^2^ and H^4^), 4.02 (2H, q, *J* 7.2, H^13^), 3.72 (2H, s, H^11^), 2.96 (12H, s, H^20^), 1.20 (6H, t *J* 7.1, H^1^), 1.14 (3H, t, *J* 7.2, H^14^). ^13^C (151 MHz, CDCl_3_): *δ*_C_ 170.9 (C^3^), 169.6 (C^12^), 151.1 (C^15^), 151.0 (C^19^), 147.2 (C^5^), 130.1 (C^17^), 129.7 (C^7^), 129.5 (C^9^), 128.5 (C^10^), 126.6 (C^8^), 122.5 (C^6^), 111.1 (C^16^), 108.8 (C^18^), 98.0 (C^21^), 61.1 (C^13^), 60.5 (C^2^), 53.7 (C^4^), 40.3 (C^20^), 34.8 (C^11^), 14.3 (C^1^), 14.2 (C^14^). ESI-LRMS *m*/*z* 648.39 ([C_35_H_42_N_3_O_7_S]^+^, 100%). ESI-HRMS calculated for [C_35_H_42_N_3_O_7_S]^+^ 648.2759, found 648.2743.

### SO-APTRA-Rosamine-Et_3_


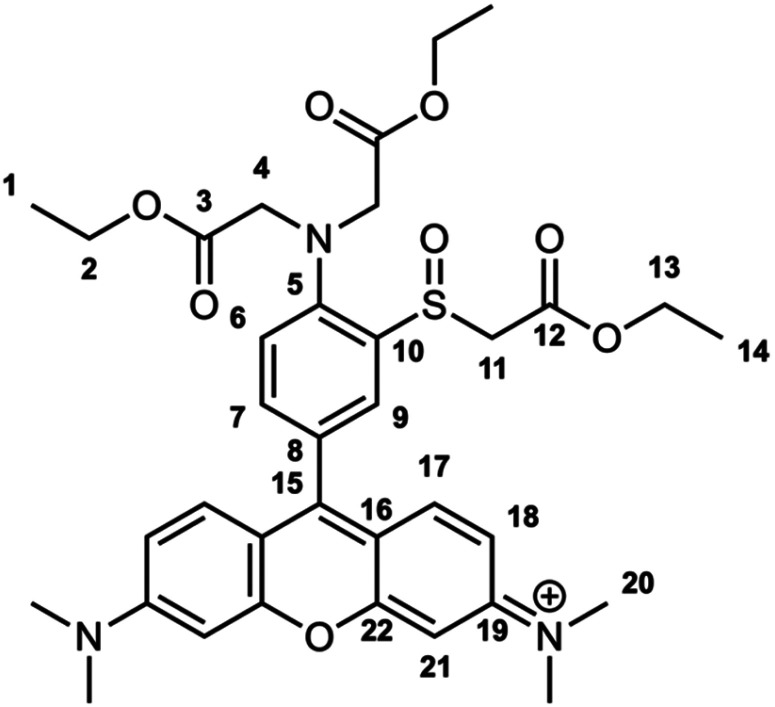
 S-APTRA-Rosamine-Et_3_ (21 mg, 0.032 mmol) was added to dry DCM (2.0 cm^3^) under a nitrogen atmosphere. The solution was cooled to 0 °C and a solution of *m*-CPBA (6.0 mg, 0.032 mmol) in DCM (0.50 cm^3^) was added slowly. The reaction mixture was stirred at RT for 1 h with a further addition of *m*-CPBA (6.0 mg, 0.032 mmol) in DCM (0.50 cm^3^) and then stirred for a further 1 h. Dilution of the reaction mixture with DCM occurred before washing with NaHCO_3_ (2 × 10 cm^3^) and brine (10 cm^3^). The organic layer was dried (MgSO_4_) with the DCM removed under reduced pressure. The bright purple solid was purified by column chromatography on silica (gradient from 100% DCM to 10% MeOH, 90% DCM) to give a mildly impure purple solid which was further purified by preparative HPLC (gradient from 10% MeCN, 90% H_2_O to 100% MeCN) to give SO-APTRA-Rosamine-Et_3_ as a purple solid (2.0 mg, 8%). ^1^H (599 MHz, CDCl_3_): *δ*_H_ 7.95–7.92 (1H, m, H^9^), 7.58–7.55 (1H, m, H^7^), 7.47–7.45 (1H, m, H^6^), 7.46–7.42 (1H, m, H^17A^), 7.36–7.33 (1H, m, H^17B^), 7.09–7.04 (1H, m, H^18A^), 6.98–6.95 (1H, m, H^18B^), 6.87–6.83 (2H, m, H^21^), 4.67–4.63 (1H, m, H^11A^), 4.37–4.31 (2H, m, H^4A^), 4.27–4.14 (6H, m, H^2^ and H^13^), 4.09–4.03 (2H, m, H^4B^), 3.90–3.86 (1H, m, H^11B^), 3.35–3.23 (12H, m, H^20^), 1.30 (6H, t, *J* 7.2, H^1^), 1.26 (3H, t, *J* 7.1, H^14^). ^13^C (151 MHz, CDCl_3_): *δ*_C_ 169.7 (C^3^), 165.3 (C^12^), 157.8 (C^22A^), 157.6 (C^22B^), 157.3 (C^19A^), 157.1 (C^19B^), 156.3 (C^15^), 148.3 (C^5^), 136.9 (C^10^), 133.3 (C^7^), 131.8 (C^17A^), 131.6 (C^17B^), 127.9 (C^8^), 126.8 (C^9^), 122.7 (C^6^), 114.8 (C^18A^), 114.4 (C^18B^), 113.5 (C^16A^), 113.3 (C^16B^), 96.9 (C^21^), 61.9 (C^13^), 61.5 (C^2^), 57.6 (C^11^), 54.5 (C^4^), 41.2 (C^20A^), 41.1 (C^20B^), 14.2 (C^1^ and C^14^). ESI-LRMS *m*/*z* 664.43 ([C_35_H_42_N_3_O_8_S]^+^, 100%). ESI-HRMS calculated for [C_35_H_42_N_3_O_8_S]^+^ 664.2692, found 664.2693.

### 4-Formyl-S-APDIA-Et_2_


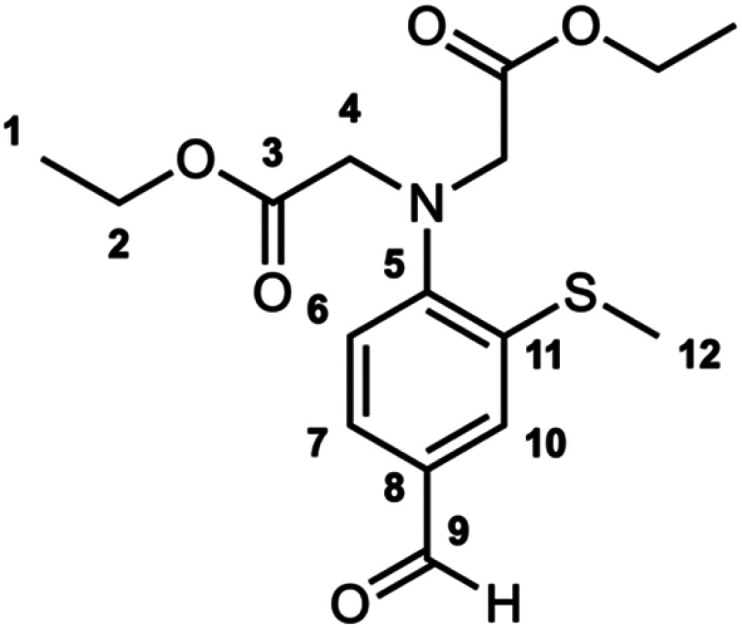
 Hexamethylenetetramine (72 mg, 0.51 mmol) was added to a solution of S-APDIA-Et_2_ (53 mg, 0.51 mmol) in TFA (2.0 cm^3^) under a nitrogen atmosphere at 0 °C. The solution was refluxed for 12 h. After cooling, the reaction was quenched using saturated aqueous NaHCO_3_ and then diluted with brine. The crude product was extracted using EtOAc (3 × 10 cm^3^) and washed with brine (10 cm^3^) before drying over MgSO_4_ and removal of the solvent under reduced pressure. The product was determined to have formed near quantitatively from S-APDIA-Et_2_*via* the ^1^H NMR spectrum meaning no further purification was attempted prior to use in subsequent steps (83 mg). ^1^H (600 MHz, CDCl_3_): *δ*_H_ 9.88 (1H, s, H^9^), 7.70–7.69 (1H, m, H^10^), 7.59–7.57 (1H, m, H^7^), 7.26–7.24 (1H, m, H^6^), 4.23 (4H, s, H^4^), 4.16 (4H, q, *J* 7.1, H^2^), 2.49 (3H, s, H^12^), 1.24 (6H, t, *J* 7.1, H^1^). ^13^C (151 MHz, CDCl_3_): *δ*_C_ 190.8 (C^9^), 170.2 (C^3^), 152.7 (C^5^), 134.3 (C^11^), 132.2 (C^8^), 128.1 (C^6^), 127.4 (C^7^), 122.1 (C^10^), 60.9 (C^2^), 53.3 (C^4^), 15.4 (C^12^), 14.4 (C^1^). ESI-LRMS *m*/*z* 339.95 ([C_16_H_21_NO_5_S + H]^+^, 100%). ESI-HRMS calculated for [C_16_H_22_NO_5_S]^+^ 340.1217, found 340.1219.

### S-APDIA-Rosamine-Et_2_


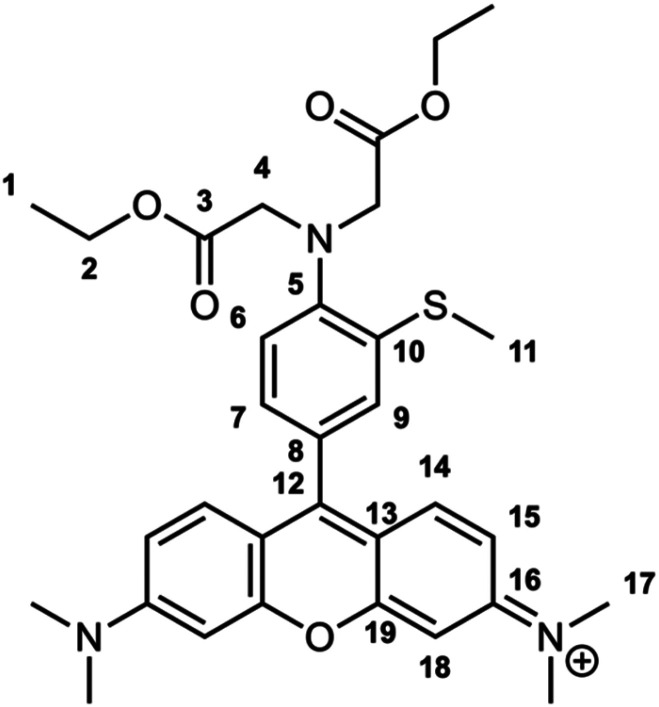
 4-Formyl-S-APDIA-Et_2_ (0.12 mg, 0.36 mmol) and 3-(dimethylamino)phenol (0.12 mg, 0.89 mmol) were combined in TCE (3.2 cm^3^) under an argon atmosphere. *p*-Toluenesulfonic acid monohydrate (0.12 mg, 0.64 mmol) was added, and the solution was heated at 80 °C for 18 h. Upon cooling, *p*-chloranil was added and then allowed to stir for a further 18 h. The reaction mixture was diluted with DCM and washed with H_2_O (3 × 10 cm^3^) and brine (10 cm^3^) before drying of the organic layer over MgSO_4_ and removal of the solvent under reduced pressure. This produced a purple solid which was purified by column chromatography on silica (gradient from 100% DCM to 90% DCM, 10% MeOH) to afford the product as a purple solid (8.0 mg, 4%). ^1^H (600 MHz, CDCl_3_): *δ*_H_ 7.44–7.40 (3H, m, H^14^ and H^9^), 7.12–7.06 (2H, m, H^6^ and H^7^), 6.99–6.96 (2H, m, H^15^), 6.87–6.85 (2H, m, H^18^), 4.26 (4H, s, H^4^), 4.20 (4H, q, *J* 7.2, H^2^), 3.36 (12H, s, H^17^), 2.41 (3H, s, H^11^), 1.28 (6H, t, *J* 7.2, H^1^). ^13^C (151 MHz, CDCl_3_): *δ*_C_ 170.6 (C^3^), 157.7 (C^19^), 157.4 (C^12^), 157.2 (C^16^), 149.1 (C^5^), 135.5 (C^10^), 127.8 (C^8^), 126.7 (C^6^ or C^7^), 126.6 (C^6^ or C^7^), 138.1 (C^14^), 123.2 (C^9^), 114.3 (C^15^), 113.4 (C^13^), 97.0 (C^18^), 61.0 (C^2^), 53.3 (C^4^), 41.2 (C^17^), 14.2 (C^11^), 14.1 (C^1^). ESI-LRMS *m*/*z* 576.30 ([C_32_H_38_N_3_O_5_S]^+^, 100%). ESI-HRMS calculated for [C_32_H_38_N_3_O_5_S]^+^ 576.2534, found 576.2532.

### SO-APDIA-Rosamine-Et_2_


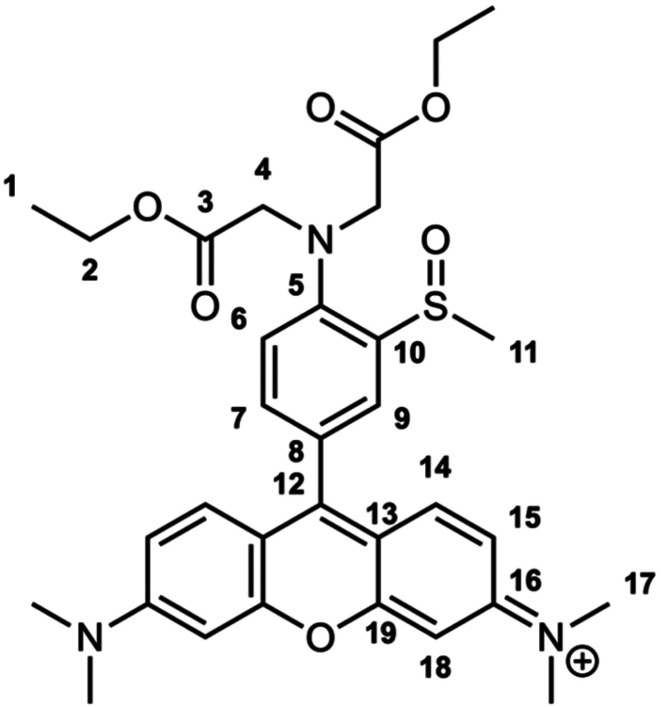
 S-APDIA-Rosamine-Et_2_ (0.13 mg, 0.23 mmol) was added to dry DCM (2.0 cm^3^) under a nitrogen atmosphere. The solution was cooled to 0 °C and a solution of *m*-CPBA (40 mg, 0.23 mmol) in DCM (0.50 cm^3^) was added slowly. The reaction mixture was stirred at RT for 1 h. Dilution of the reaction mixture with DCM occurred before washing with NaHCO_3_ (2 × 10 cm^3^) and brine (10 cm^3^). The organic layer was dried (MgSO_4_) with the DCM removed under reduced pressure. The bright purple solid was purified by preparative HPLC (gradient from 10% MeCN, 90% H_2_O to 100% MeCN) to give SO-APDIA-Rosamine-Et_2_ as a purple solid (87 mg, 63%). ^1^H (599 MHz, CDCl_3_): *δ*_H_ 7.95–7.92 (1H, m, H^9^), 7.50–7.47 (1H, m, H^7^), 7.46–7.36 (1H, m, H^6^), 7.40–7.37 (1H, m, H^14A^), 7.36–7.31 (1H, m, H^14B^), 7.17–7.14 (1H, m, H^15A^), 6.95–6.92 (1H, m, H^15B^), 6.81 (2H, s, H^18^), 4.36–4.30 (2H, m H^4A^), 4.23–4.16 (4H, m, H^2^), 4.09–4.03 (2H, m, H^4B^), 3.37 (6H, s, H^17A^), 3.33 (6H, s, H^17B^), 2.98 (3H, s, H^11^), 1.28 (6H, t, *J* 7.3, H^1^). ^13^C (151 MHz, CDCl_3_): *δ*_C_ 169.8 (C^3^), 157.8 (C^19A^), 157.6 (C^19B^), 157.4 (C^16A^), 157.1 (C^16B^), 156.4 (C^12^), 148.5 (C^5^), 140.3 (C^10^), 132.9 (C^7^), 131.7 (C^14^), 127.9 (C^8^), 125.8 (C^9^), 122.7 (C^6^), 115.2 (C^15A^), 114.3 (C^15B^), 113.4 (C^13A^), 113.5 (C^13B^), 96.8 (C^18^), 61.4 (C^2^), 54.4 (C^4^), 41.1 (C^17A^), 41.1 (C^17B^), 14.3 (C^1^). ESI-LRMS *m*/*z* 592.75 ([C_32_H_38_N_3_O_6_S]^+^, 100%). ESI-HRMS calculated for [C_32_H_38_N_3_O_6_S]^+^ 592.2471, found 592.2481.

## Conflicts of interest

The authors have no conflicts of interest to declare.

## Supplementary Material

OB-024-D6OB00551A-s001

## Data Availability

The data supporting this article have been included as part of the supplementary information (SI). Supplementary information: synthetic details for intermediates; NMR spectra of all new materials; additional absorption, emission, and excitation spectra; further details of fitting procedures for fluorescence tritrations; details of cell imaging experiments; cytotoxicity data. See DOI: https://doi.org/10.1039/d6ob00551a.
