# Supplementary material for: Rosamine derivatives of o-aminothiophenol-triacetate (S-APTRA): a new class of selective fluorescent sensors for Zn2+
Source: Org Biomol Chem. 2026 Jul 7;24(28):5875–88. doi: 10.1039/d6ob00551a (PMC13340082; doi:10.1039/d6ob00551a)
Supplement: OB-024-D6OB00551A-s001 [file OB-024-D6OB00551A-s001.pdf]

Supplementary Information (SI) for Organic & Biomolecular Chemistry.
This journal is © The Royal Society of Chemistry 2026

SUPPORTING INFORMATION

Rosamine derivatives of *o*-aminothiophenol-triacetate (S-APTRA): A new class of selective fluorescent sensors for Zn^{2+}

Laura L. Duncan*, Dominic J. Black, Robert Pal, and J. A. Gareth Williams*

Department of Chemistry, Durham University, Durham, DH1 3LE, U.K.

E-mail: laura.l.duncan@durham.ac.uk j.a.g.williams@durham.ac.uk

Contents

1. Synthetic Procedures and Details for Intermediates 2
2. Optical Spectroscopy Methods and Data Analysis 5
3. Excitation and Absorption Spectra 7
4. S-APTRA-Rosamine 8
4.1 Metal Ion Addition: Excitation and Absorption Spectra 8
4.2 Job Plot 9
4.3 Mg^{2+} Fluorescence Titration 9
4.4 Metal Ion Screen 9
5. S-APDIA-Rosamine 10
5.1 Absorption and Emission Spectra 10
5.2 Metal Ion Addition: Emission Spectra 10
5.3 Metal Ion addition: Excitation and Absorption Spectra 11
6. SO-APTRA-Rosamine 12
6.1 Metal Ion Addition: Excitation and Absorption Spectra 12
6.2 Metal Ion Additon: Emission Spectra 13
7. SO-APDIA-Rosamine 14
7.1 Metal Ion Addition: Emission Spectra 14
7.2 Metal Ion Addition: Absorption and Excitation Spectra 14
8. Cell Cultures, Microscopy and Imaging 15
8.1 Cytotoxicity Assays 16
8.2 Transmission Images 17
9. ^{1}H and $^{13}C\{^{1}H\}$ NMR Spectra 19
10. ESI-LRMS Spectra 25
11. References 27

1. Synthetic Procedures and Details for Intermediates

All chemicals were acquired from chemical suppliers and used without further purification. Both air- and moisture-sensitive reactions were carried on under a nitrogen or argon atmosphere using a Schlenk line. Silica column chromatography was performed on combi-flash instruments using RediSep R_f silica cartridges. All thin layer chromatography was carried out on silica plates (Merck Art 5554) using UV irradiation (254 nm or 366 nm). ^1H and $^{13}C\{^1H\}$ NMR spectra, including two-dimensional spectra, were recorded on either a Bruker Avance III-HD-400 spectrometer, Bruker Neo-400 spectrometer, Bruker Neo-700 spectrometer or a Varian VNMRS-600 spectrometer. Electrospray mass spectra were recorded on a Waters SQD system interfaced with an Acquity UPLC system.

S-APTRA-Et$_3$

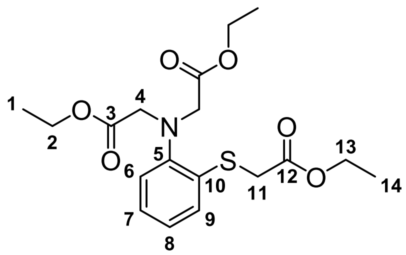

2-Aminothiophenol (1.0 cm^3, 10 mmol) and chloroacetic acid (4.7 g, 50 mmol) were combined in a NaOH solution (7 M, 10 cm^3). The reaction was heated at reflux for 4 h with the pH monitored every 15 min and NaOH pellets added if the pH dropped < 10. The reaction mixture was left to cool to room temperature, and the solvent was removed under reduced pressure. EtOH (35 cm^3) and concentrated sulfuric acid (4.2 cm^3) were added, and the reaction mixture was heated at reflux for 3 d. The solvent was removed under reduced pressure with EtOAc added to the resulting crude product and then filtered to remove the inorganic impurities. The EtOAc solution was then washed with NaOH solution (10 %, 10 cm^3) and H_2O (10 cm^3) before drying ($MgSO_4$) and removal of the solvent under reduced pressure. Purification was achieved via column chromatography on silica (a gradient from 100 % PET ether to 50 % PET ether, 50 % EtOAc) followed by reverse-phase column chromatography on silica (gradient from 10 % MeCN, 90 % H_2O to 100 % MeCN). This yielded a yellow oil (0.45 g, 12 %). ^1H (400 MHz, $CDCl_3$): δ_H 7.37 – 7.32 (1H, m, H^9), 7.25 – 7.20 (1H, m, H^6), 7.20 – 7.14 (1H, m, H^7), 7.04 – 6.96 (1H, m, H^8), 4.22 (4H, s, H^4), 4.20 – 4.10 (6H, m, H^2 and H^{13}), 3.79 (2H, s, H^{11}), 1.28 – 1.18 (9H, m, H^1 and H^{14}). ESI-LRMS *m/z* 384.27 ($[C_{18}H_{25}NO_6S + H]^+$, 100 %). Spectra consistent with the literature data.[1]

2-Nitrothiophenol

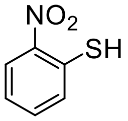

2-Nitrophenol disulfide (0.50 g, 1.6 mmol), PPh_3 (0.60 g, 2.4 mmol) and 2-mercaptoethanol (0.12 cm^3, 1.6 mmol) were combined in THF (13 cm^3) and H_2O (0.30 cm^3). The resulting yellow solution was heated at 50 °C for 16 h. The reaction mixture was allowed to cool to RT before removal of the solvent under reduced pressure. The crude product was dissolved in DCM and washed with brine (3 × 10 cm^3) before drying ($MgSO_4$) and removal of the solvent under reduced pressure. Product purification was achieved using column chromatography on silica (gradient from 100 % PET ether to 10 % EtOAc, 90 % PET ether) to give a yellow solid (0.23 g, 91 %). ^1H (400 MHz, $CDCl_3$): δ_H 8.27 – 8.22 (1H, m, Ar), 7.47 – 7.44 (2H, m, Ar), 7.33 – 7.27 (1H, m, Ar), 4.04 (1H, s, SH). Spectra are consistent with literature data.[1]

Methyl(2-nitrophenyl)sulfane

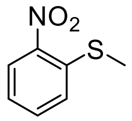

2-Nitrothiophenol (0.23 g, 1.5 mmol) and K_2CO_3 (0.59 g, 4.5 mmol) were combined in anhydrous MeCN (4.0 cm^3) under argon. Methyl iodide (0.10 cm^3, 1.6 mmol) was added and the reaction was stirred at RT for 2 h. The solvent was removed under reduced pressure with the resulting crude product dissolved in DCM and filtered. This gave the final product as a bright yellow solid (0.21 g, 83 %). ^1H (400 MHz, $CDCl_3$): δ_H 8.22 – 8.15 (1H, m, Ar), 7.57 – 7.48 (1H, m, Ar), 7.34 – 7.28 (1H, m, Ar), 7.23 – 7.15 (1H, m, Ar), 2.43 (3H, s, CH_3). Spectra are consistent with literature data.[1]

2-(Methylthio)aniline

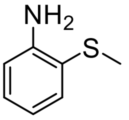

Methyl(2-nitophenyl)sulfane (1.0 g, 6.1 mmol) was dissolved in EtOH (7.0 cm^3). NH_4Cl (0.81 g, 15 mmol), iron powder (1.7 g, 30 mmol) and H_2O (7.0 cm^3) were added, and the reaction mixture was heated at 70 °C for 2 h with complete conversion of the starting material to product confirmed via TLC with the use of a ninhydrin stain. The reaction mixture was filtered hot through celite and washed with hot EtOH before removal of the solvents under reduced pressure. The resulting crude product was dissolved in DCM and washed with H_2O (3 × 10 cm^3) before drying over $MgSO_4$ and removal of the solvent under reduced pressure to give a yellow oil (0.45 g, 54 %). ^1H (400 MHz, $CDCl_3$): δ_H 7.50 – 7.36 (1H, m, Ar), 7.14 – 7.10 (1H, m, Ar), 6.77 – 6.72 (2H, m, Ar), 4.28 (2H, s, br, NH_2), 2.40 (3H, s, CH_3). Spectra are consistent with literature data.[1]

S-APDIA-Et_2

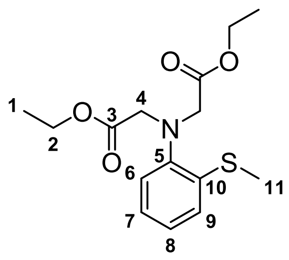

2-(Methylthio)aniline (45 mg, 3.2 mmol), DIPEA (2.8 cm^3, 16 mmol), ethyl bromoacetate (1.8 cm^3, 16 mmol) and KI (0.54 mg, 3.2 mmol) were combined in anhydrous MeCN (10 cm^3) under argon. The reaction was refluxed at 85 °C for 2 d. Additional DIPEA (1.4 cm^3, 8.1 mmol) and ethyl bromoacetate (0.90 cm^3, 8.1 mmol) were added and the reaction mixture was refluxed for a further 3 d. The MeCN was removed under reduced pressure, and the crude product was dissolved in EtOAc and filtered to remove the inorganic impurities. The filtrate was washed with H_2O (10 cm^3) and brine (10 cm^3) before drying over $MgSO_4$ and removal of the EtOAc under reduced pressure. Purification was achieved using column chromatography on silica (gradient from 100 % PET ether to 90 % PET ether, 10 % EtOAc) to give the resulting product as a yellow oil (0.11 g, 4 %). ^{1}H (400 MHz, $CDCl_3$): δ_H 7.26 – 7.24 (1H, m, H^9), 7.13 – 7.09 (1H, m, H^7), 7.07 – 7.04 (2H, m, H^6 and H^8), 4.15 – 4.05 (8H, m, H^2 and H^4), 2.40 (3H, s, H^{11}), 1.20 (6H, t, J 7.2, H^1). Spectra are consistent with literature data.[1]

Hydrolysis of the tris- and bis-ethyl esters to generate the carboxylate ligands

The ester was added to a solution of MeOH (0.1 cm^3 per 5.0 mg of ester) and aqueous NaOH (0.1 cm^3 per 5.0 mg of ester). The solution was stirred at RT for 1 h with complete hydrolysis confirmed using ESI-LRMS. The solution was diluted to 5 cm^3 using aqueous buffer (50 mM HEPES, 100 mM KCl, pH 7.2). Aliquots of the 5 cm^3 solution were then used to form solutions of known ligand concentration.

S-APTRA-Rosamine – from S-APTRA-Rosamine-Et_3 (2.6 mg). ESI-LRMS *m/z* 564.40 ($[C_{29}H_{30}N_3O_7S]^+$, 100 %). ESI-HRMS calculated for $[C_{29}H_{30}N_3O_7S]^+$ 564.1803, found 564.1804.

SO-APTRA-Rosamine – from SO-APTRA-Rosamine-Et_3 (2.6 mg). ESI-LRMS *m/z* 580.21 ($[C_{29}H_{30}N_3O_8S]^+$, 100 %). ESI-HRMS calculated for $[C_{29}H_{30}N_3O_8S]^+$ 580.1772, found 580.1754.

S-APDIA-Rosamine – from S-APDIA-Rosamine-Et_2 (4.1 mg). ESI-LRMS *m/z* 520.47 ($[C_{28}H_{30}N_3O_5S]^+$, 100 %). ESI-HRMS calculated for $[C_{28}H_{30}N_3O_5S]^+$ 520.1915, found 520.1940.

SO-APDIA-Rosamine – from SO-APDIA-Rosamine-Et_2 (14.7 mg). ESI-LRMS *m/z* 536.39 ($[C_{28}H_{30}N_3O_6S]^+$, 100 %). ESI-HRMS calculated for $[C_{28}H_{30}N_3O_6S]^+$ 536.1853, found 536.1855.

2. Optical Spectroscopy Methods and Data Analysis

General information

Absorbance measurements were acquired with a UVIKON XS spectrometer or an Agilent Cary 5000. Quartz cuvettes of 1 cm pathlength were used, with spectra collected in 1 nm increments at a rate of 200 nm / min, recorded against the buffer solution in an optically matched cuvette. Fluorescence spectra were recorded using a FluoroLog-3 spectrometer equipped with a Hamamatsu R928 detector. A quartz cuvette was used with a pathlength of 1 cm. Spectra were acquired at 1 nm increments with an integration time of 0.5 s. Quantum Yields were determined using rhodamine b isothiocyanate in EtOH as the standard ($\Phi = 0.65$)[2]. Fluorescence lifetimes were determined by time-correlated single-photon counting (TCSPC) using an Edinburgh Instruments OB920 spectrometer following excitation at 374 nm with a pulsed laser diode.

Spectrophotometric titrations and determination of K_d

All binding studies were carried out in an aqueous buffer system (50 mM HEPES, pH 7.2, 100 mM KCl). In each case, the titrations were repeated at least three times with the quoted uncertainty being the standard error on the mean.

Mg^{2+}, Ca^{2+}, and Zn^{2+} when K_d in μM range or above. Aliquots of the metal ion solution were added to a solution of the ligand (2 cm^3). The metal solutions also contained the same concentration of the ligand to avoid issues with ligand dilution over the course of the titration. After the addition of each aliquot, the solution was mixed via inversion of the cuvette and left to equilibrate for 5 min before acquisition of the emission spectrum. Aliquots were added until 1 cm^3 total of the metal solution had been added to achieve metal saturation conditions. The emission intensity at a specific wavelength showing a suitable progressive change over the course of the titration was chosen and fitted using a 1:1 binding model from www.supramolecular.org[3]

Zn^{2+} when K_d in sub-μM range.[1] To a solution of the ligand (2 cm^3), also containing ethylene glycol-bis(*β*-aminoethyl ether)-*N*,*N*,*N'*,*N'*-tetraacetic acid (EGTA, 1 mM), aliquots of the Zn^{2+} solution were gradually added to the cuvettes. As above, the metal solutions also contained the same concentration of the ligand, to avoid issues with ligand dilution over the course of the titration, but in this case EGTA too (1 mM). After the addition of each aliquot, the solution was mixed via inversion of the cuvette and left to equilibrate for 5 min before acquisition of the emission spectrum. Aliquots were added until 1 cm^3 total of the metal solution had been added to achieve metal saturation conditions (total Zn^{2+} = 1 mM). The concentration of free Zn^{2+} was calculated using Equation 1 taking the value of $K_{d(EGTA)}$ to be 3.78×10^8 M^{-1}.[4] The value of K_d for the ligand was then determined via a non-linear least squares iterative analysis using Equation 2.

$$K_{d(EGTA)}([Zn^{2+}]_{free})^2 + (1 + K_{d(EGTA)}[L] - K_{d(EGTA)}[Zn^{2+}]_{total})([Zn^{2+}]_{free}) - [Zn^{2+}]_{total} = 0 \quad \textbf{(1)}$$

$$A = \frac{A_{min} + A_{max}K_a[Zn^{2+}]_{free}}{1 + K_a[Zn^{2+}]_{free}} \quad \textbf{(2)}$$

Determination of pK_a values by pH titrations

The pH of each solution was recorded using a Jenway 924 005 pH electrode connected to a Jenway 3510 pH meter. The system was calibrated using commercial buffer solutions at pH 4.01 and 9.21. A solution of the ligand was prepared containing 100 mM KCl to ensure a roughly constant ionic strength, and aliquots of aqueous HCl (0.025 M, 0.05 M and 0.1 M) were added to shift the pH from basic to acidic. Sigmoidal curves of emission intensity against pH were fitted by non-linear least squares iteration using Equation 3.

$$A_{obs} = \frac{A_{min}10^{-pH} + K_aA_{max}}{10^{-pH} + K_a} \quad \textbf{(3)}$$

3. Excitation and Absorption Spectra

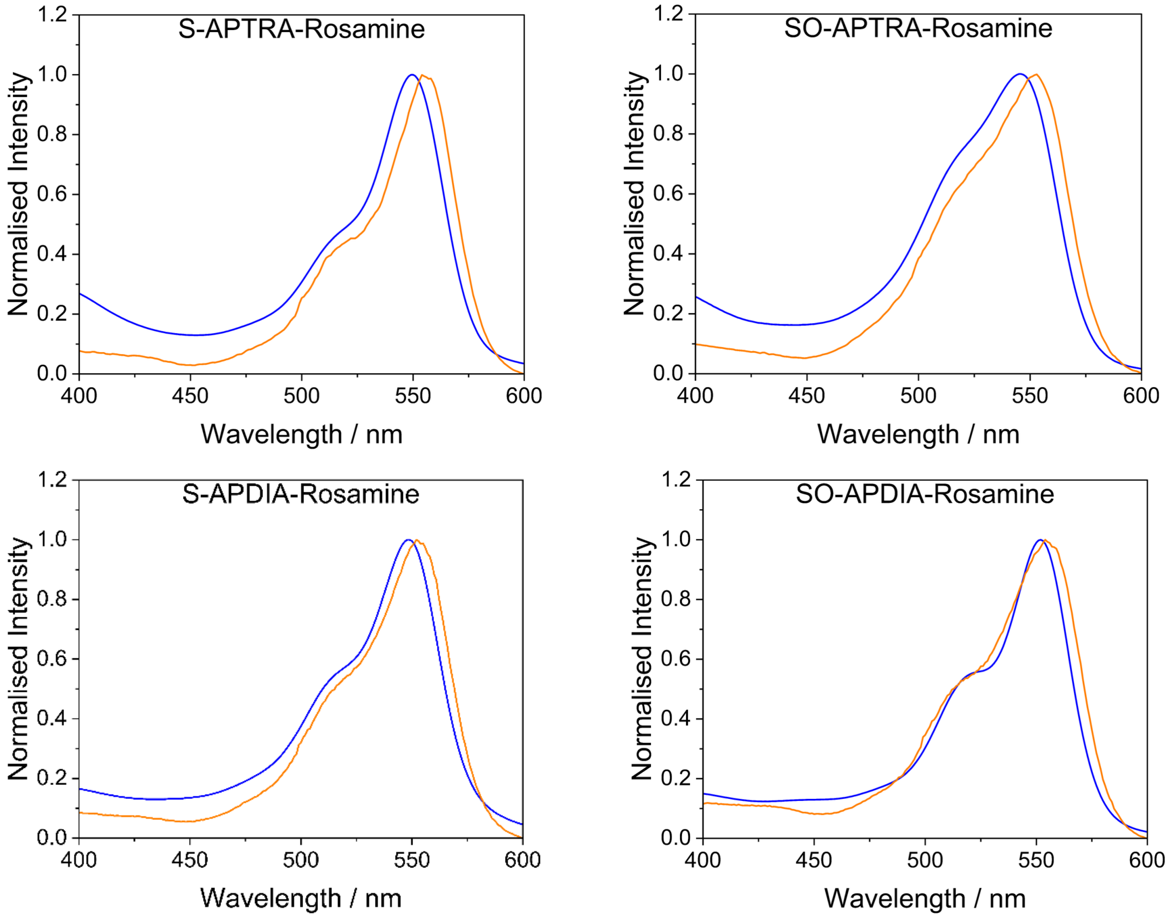

Figure S1. *Comparison of the normalised absorption (blue) and excitation (orange) spectra of the four ligands (10 µM) as indicated in the four graphs; in buffered aqueous solution (50 mM HEPES, pH 7.2, 100 mM KCl).*

4. S-APTRA-Rosamine

4.1 Metal Ion Addition: Excitation and Absorption Spectra

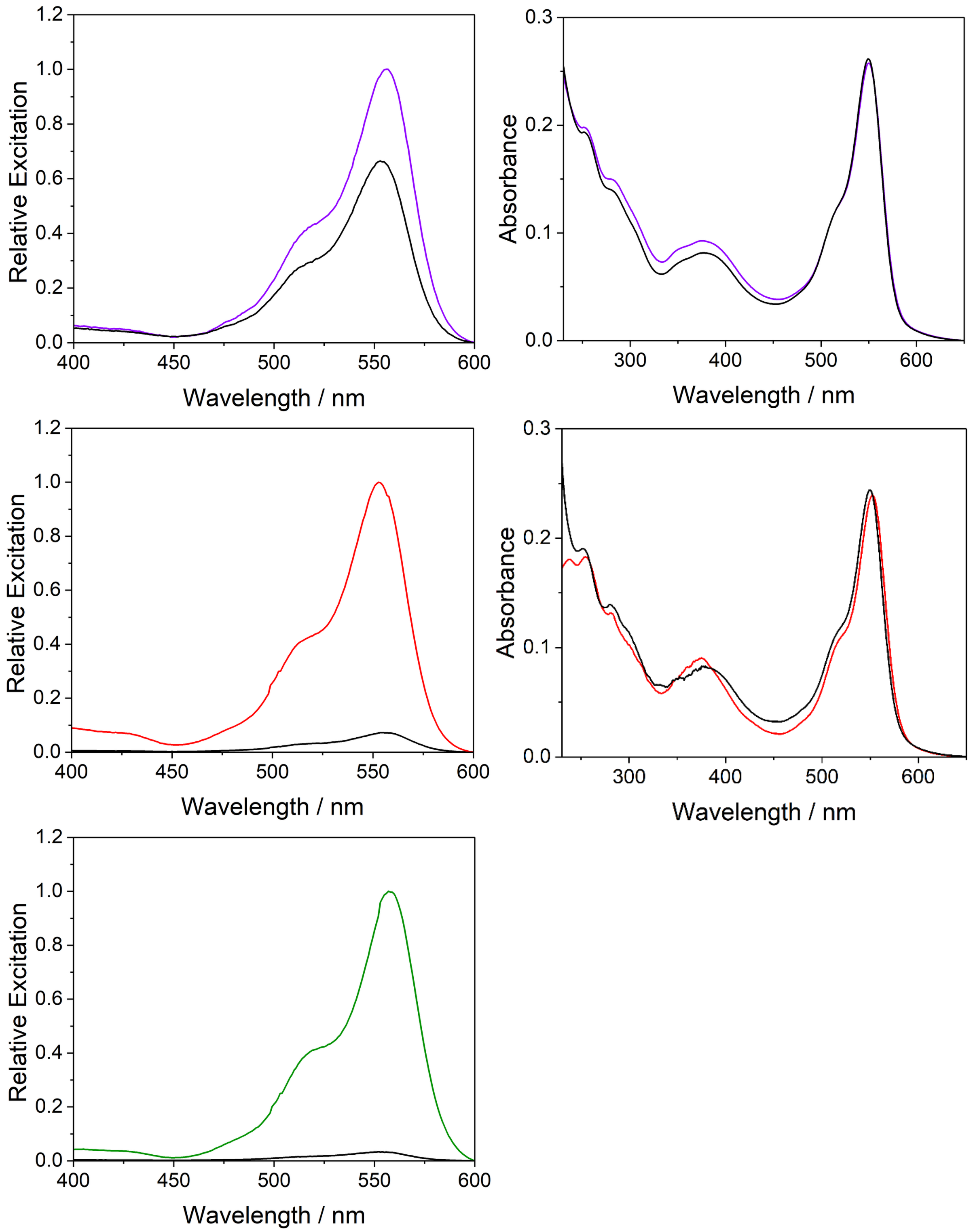

Figure S2. *Excitation spectra (left, λ_{em} = 620 nm) and absorption spectra (right) of S-APTRA-Rosamine (10 µM, black line) upon the addition of $MgCl_2$ (67 mM, purple), $CaCl_2$ (3 mM, red), and $ZnSO_4$ (1 mM, 1 mM EGTA, green). In buffered aqueous solution (50 mM HEPES, pH 7.2, 100 mM KCl).*

4.2 Job Plot

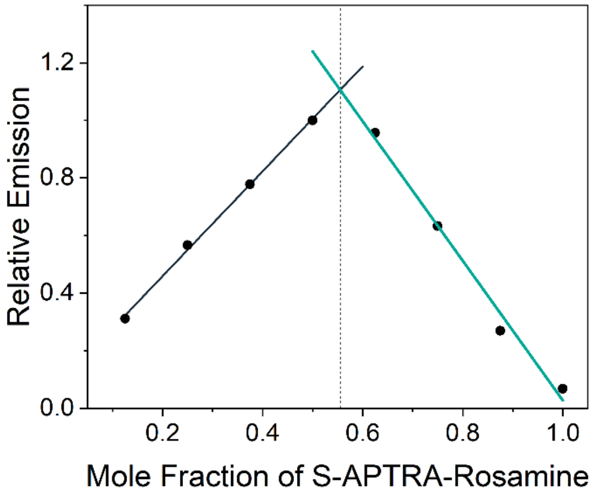

Figure S3. *Job plot of S-APTRA-Rosamine and* $ZnSO_4$ *suggesting 1:1 binding stoichiometry; in buffered aqueous solution (50 mM HEPES, pH 7.2, 100 mM KCl).*

4.3 Mg^{2+} Fluorescence Titration

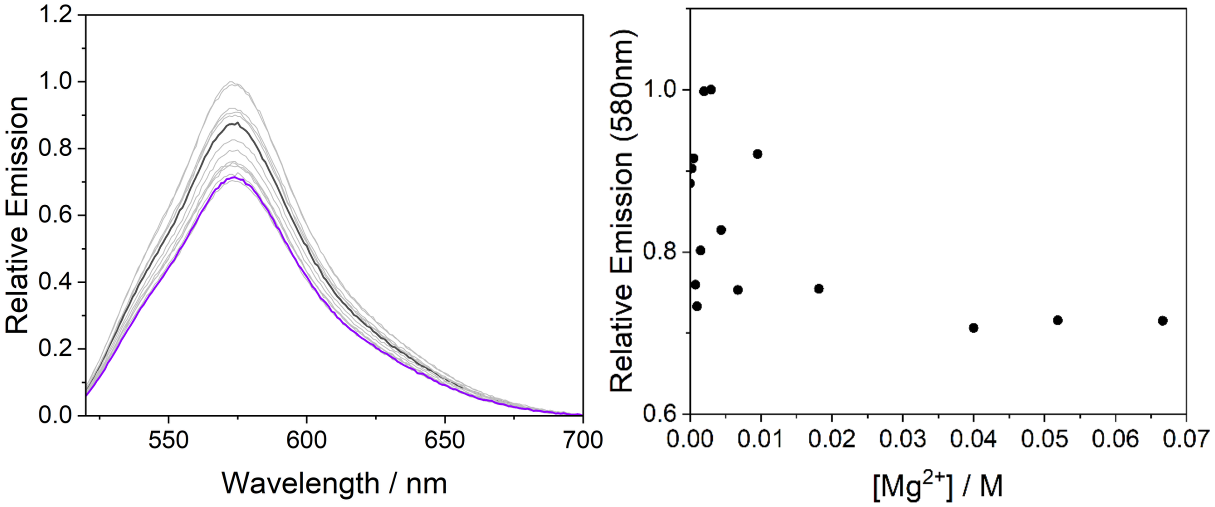

Figure S4. *Representative fluorescence titration data and associated emission at 580 nm for the addition of* $MgCl_2$ *to S-APTRA-Rosamine (10 µM). The black line is the spectrum of the metal-free ligand, and the purple that at the end of the* $MgCl_2$ *addition. In buffered aqueous solution (50 mM HEPES, pH 7.2, 100 mM KCl).*

4.4 Metal Ion Screen

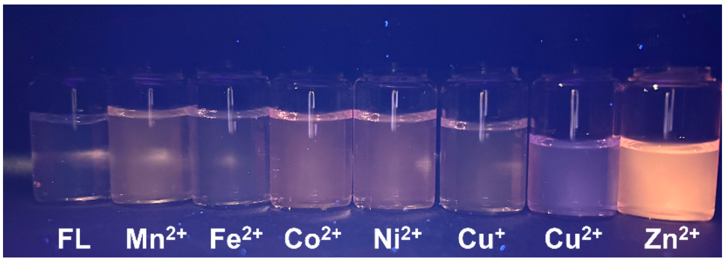

Figure S5*. Image of solutions of S-APTRA-Rosamine (10 µM) containing ions of the first-row transition metals indicated (50 µM) under UV irradiation; in buffered aqueous solution (50 mM HEPES, pH 7.2, 100 mM KCl).*

5. S-APDIA-Rosamine

5.1 Absorption and Emission Spectra

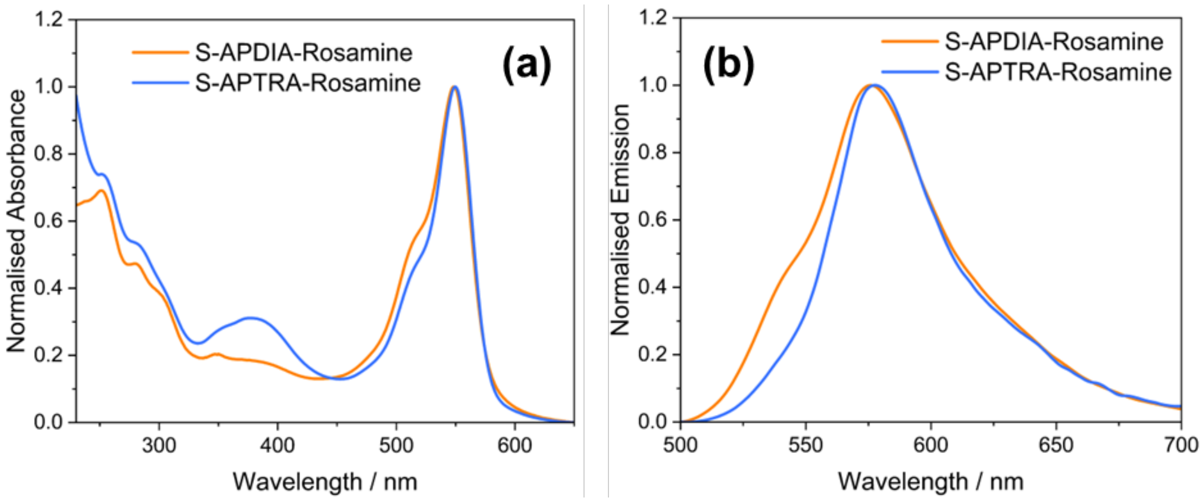

Figure S6. *(a) Absorption spectra of S-APTRA-Rosamine (blue) and S-APDIA-Rosamine (orange) at 295 K in buffered aqueous solution (50 mM HEPES, pH 7.2, 100 mM KCl). (b) Corresponding emission spectra under the same conditions, λex = 480 nm.*

5.2 Metal Ion Addition: Emission Spectra

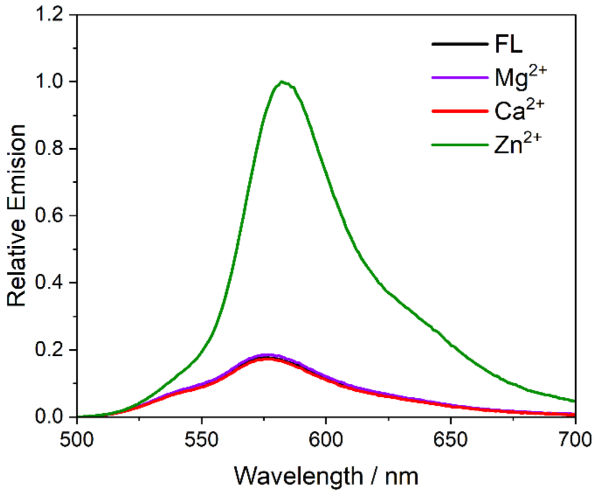

Figure S7. *Emission spectrum of S-APDIA-Rosamine (10 µM, black line) upon the addition of $MgCl_2$ (67 mM, purple), $CaCl_2$ (10 mM, red) or $ZnSO_4$ (2 mM, green); in buffered aqueous solution (50 mM HEPES, pH 7.2, 100 mM KCl).*

5.3 Metal Ion addition: Excitation and Absorption Spectra

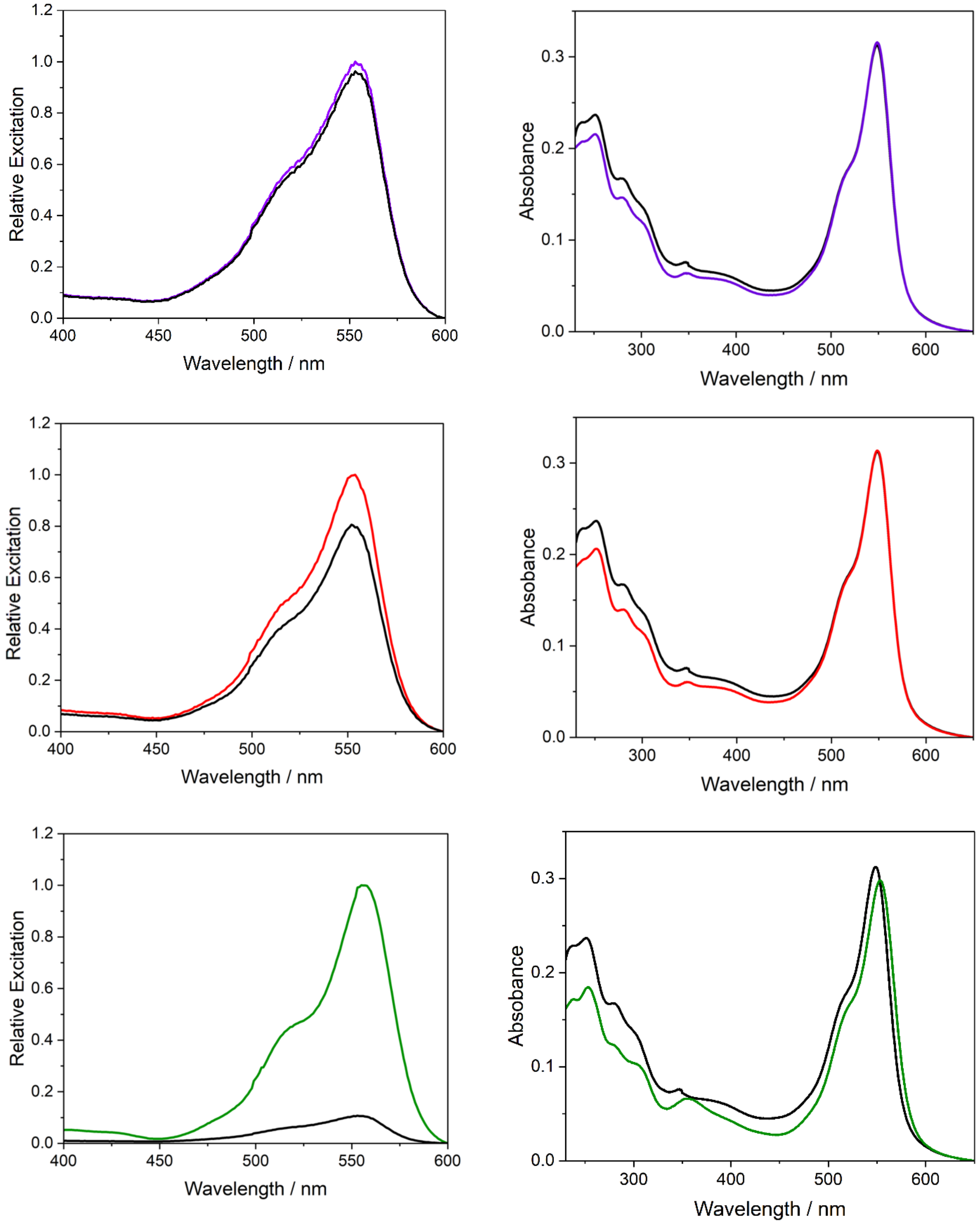

Figure S8. *Absorption (left) and excitation spectrum (right, λ_{em} = 620 nm) of S-APDIA-Rosamine (10 µM, black) upon the addition of $MgCl_2$ (67 mM, purple), $CaCl_2$ (10 mM, red) and $ZnSO_4$ (2 mM, green); in buffered aqueous solution (50 mM HEPES, pH 7.2, 100 mM KCl).*

6. SO-APTRA-Rosamine

6.1 Metal Ion Addition: Excitation and Absorption Spectra

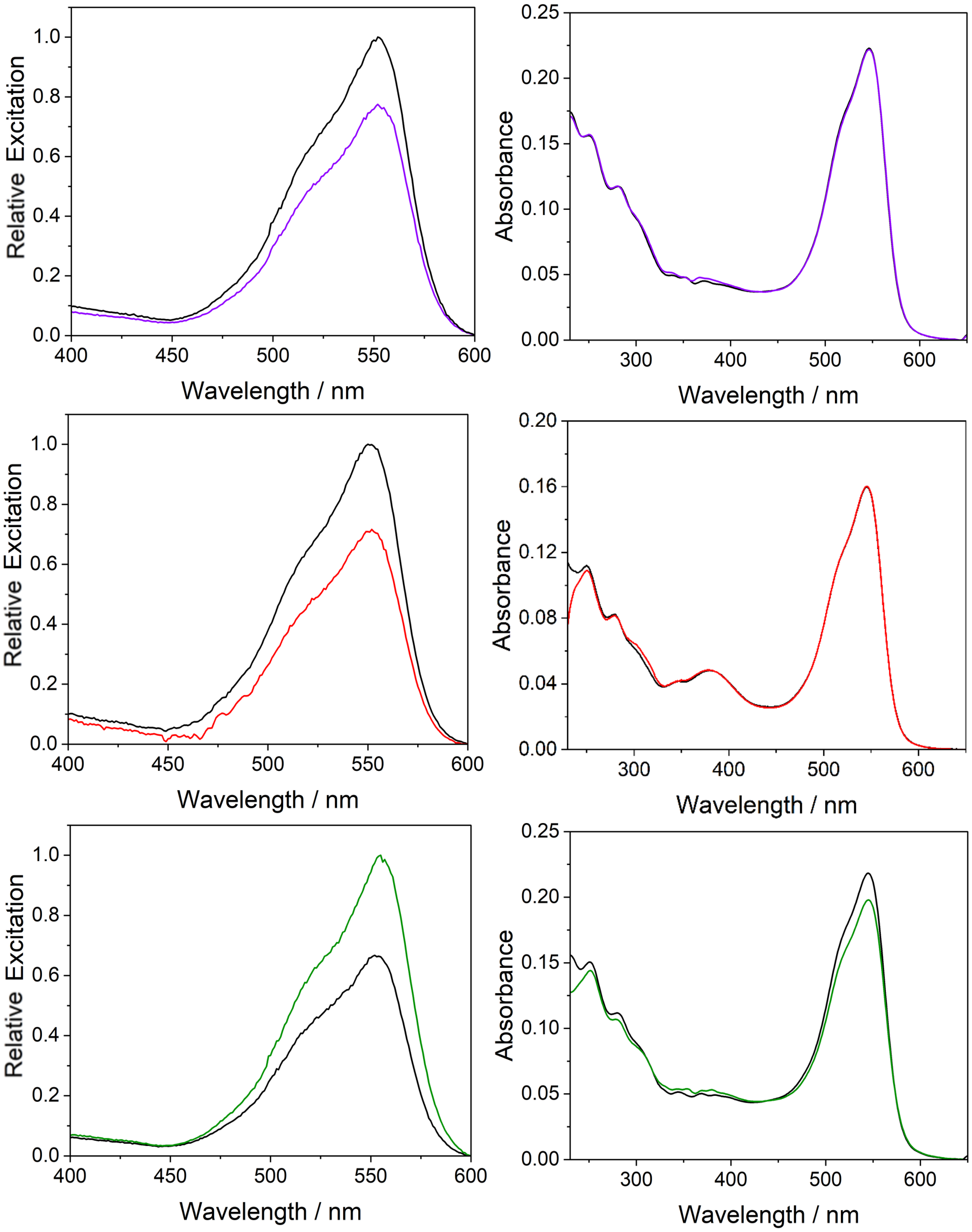

Figure S9. *Excitation spectra (left, λ_{em} = 620 nm) and absorption spectra (right) of SO-APTRA-Rosamine (10 µM, black line) upon the addition of $MgCl_2$ (67 mM, purple, top), $CaCl_2$ (667 µM, red, middle), and $ZnSO_4$ (667 µM Zn^{2+}, green, bottom); in buffered aqueous solution (50 mM HEPES, pH 7.2, 100 mM KCl).*

6.2 Metal Ion Additon: Emission Spectra

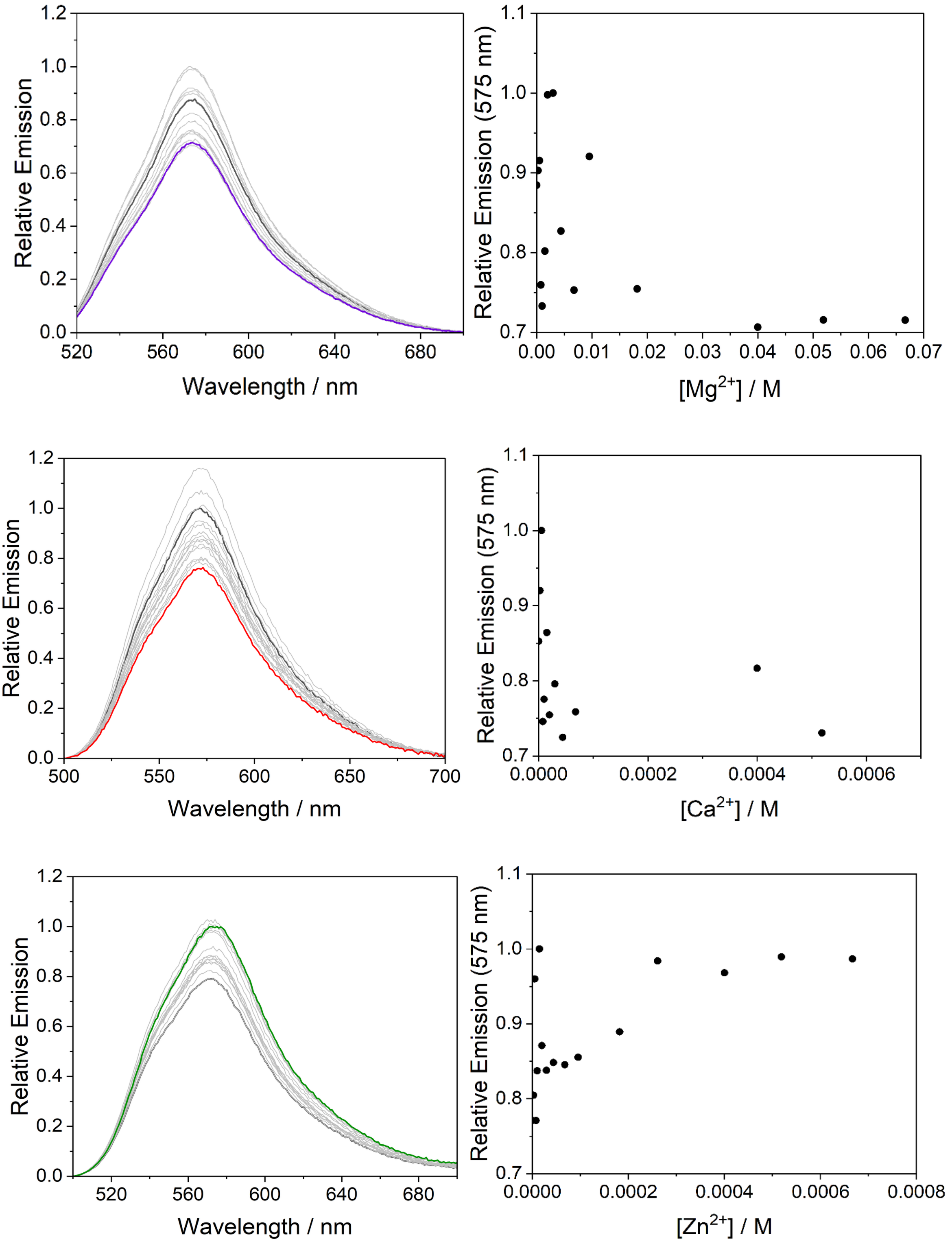

Figure S10. *Representative fluorescence titration data (λ_{ex} = 480 nm) and associated emission at 575 nm for the binding of $MgCl_2$ (top, purple), $CaCl_2$ (middle, red), or $ZnSO_4$ (bottom, green) to SO-APTRA-Rosamine (10 µM). The black spectrum is that of the metal-free ligand in each case, whereas the coloured spectrum is that at the end of the respective titration; in buffered aqueous solution (50 mM HEPES, pH 7.2, 100 mM KCl).*

7. SO-APDIA-Rosamine

7.1 Metal Ion Addition: Emission Spectra

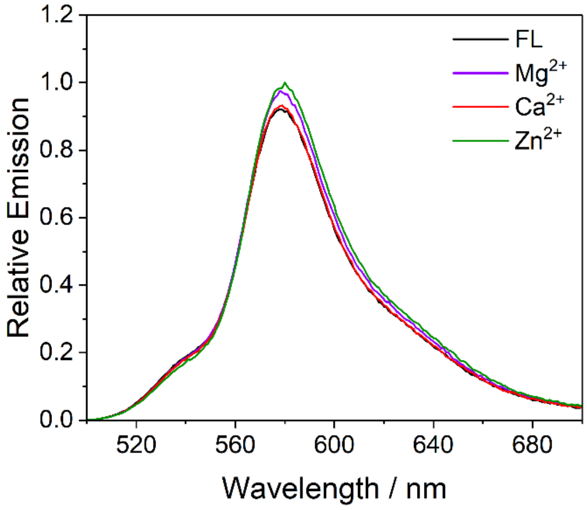

Figure S11. *Emission spectrum of SO-APDIA-Rosamine (10 µM, black line) upon the addition of $MgCl_2$ (67 mM, purple), $CaCl_2$ (10 mM, red) and $ZnSO_4$ (2 mM, green); λ_{ex} = 480 nm; in buffered aqueous solution (50 mM HEPES, pH 7.2, 100 mM KCl).*

7.2 Metal Ion Addition: Absorption and Excitation Spectra

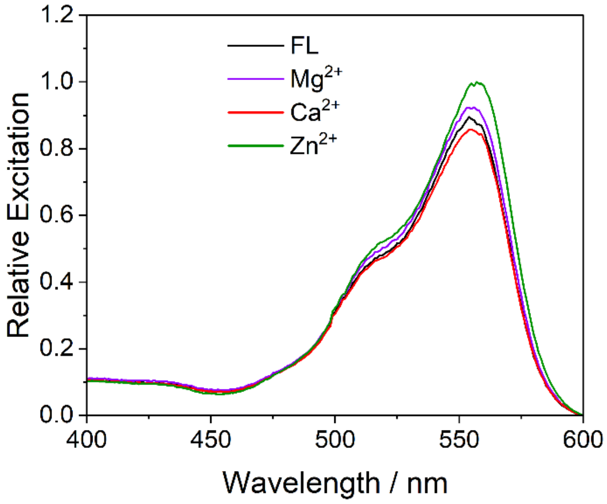

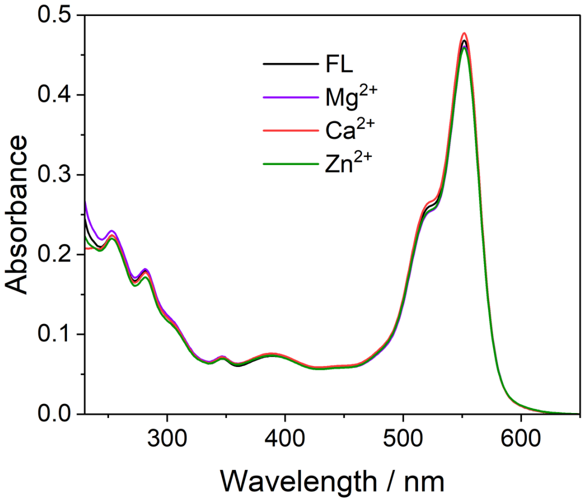

Figure S12. *Excitation (left, λ_{em} = 620 nm) and absorption spectrum (right) of SO-APDIA-Rosamine (black) upon the addition of $MgCl_2$ (67 mM, purple) and $CaCl_2$ (10 mM, red) and $ZnSO_4$ (2 mM, green). [SO-APDIA-Rosamine] = 10 µM; in buffered aqueous solution (50 mM HEPES, pH 7.2, 100 mM KCl).*

8. Cell Cultures, Microscopy and Imaging

The NIH 3T3 cell line (embryonic mouse skin fibroblast cells) was used. Cells were maintained as monolayers in F-12/DMEM (Dulbecco's Modified Eagle Medium) 1:1, supplemented with 10 % foetal bovine serum (FBS). Cells were grown in 75 cm^3 plastic culture flasks, with no prior surface treatment. Cell cultures were incubated at 37˚C, 10 % average humidity, and 5 % CO_2 v/v. Cells were harvested by treatment with trypsin solution, 0.25 % v/v for 5 min at 37˚C.

Cells were grown on ibidi 2 well μ-Slides to around 80 % confluency before dosing with S-APTRA-Rosamine and incubating for the times indicated. A 2 µM stock solution of the probe molecule in F-12/DMEM 1:1 was prepared from a 1 mM stock solution in aqueous buffer (50 mM HEPES, pH 7.2, 100 mM KCl). The staining media was carefully removed by pipetting, and fresh media without the probe was added. For experiments where Zn^{2+} and TPEN were added, a single slide was used which was removed from the microscope after the initial image was acquired, the media carefully removed by pipetting, and fresh media with the Zn^{2+} or TPEN supplemented to it was then added. The slide was returned to the microscope and images were recorded.

Steady-state fluorescence images were recorded using the PhMoNa enhanced Leica SP5 II LSCM confocal microscope[5] equipped with a HCX PL APO 63×/1.40 NA LambdaBlue Oil immersion objective. Data were collected using ×2 digital magnification at 100 Hz/line scan speed (4-line average, bidirectional scanning) at 543 nm (HeNe) laser, set at 30 mW, 400 nJ/voxel total dwell time). To achieve excitation with maximal probe emission, the microscope was equipped with a triple-channel imaging detector, comprising a conventional PMT system and two HyD hybrid avalanche photodiode detectors. The frame size was determined at 1024 × 1024 pixel, with ×2 digital magnification to ensure illumination flatness of field and 1 airy disc unit determining the applied pinhole diameter rendering on voxel to correspond to 96 × 96 nm^2 (frame size 98 × 98 μm^2 with a section thickness set at 768 nm (at 543 nm excitation). An Ar ion laser at 488 nm was used to aid parallel transmission image capture of the co-stain signals using LysoTracker™ Green DND-26 and BioTracker™ 488 Green Lipid Droplet Dye. As with previous experiments, the media containing the co-stain was removed prior to imaging. All imaging parameters are kept constant across experiments. This includes voxel size, laser power, line speed, and averaging sequences, unless otherwise noted.

All post image processing was carried out on the open source, plugin prepacked, FIJI (ImageJ 1.52p Java 1.8.0_172 64 Bit). All adjustments to voxel brightness and contrast were kept at constant values within each image set. Pearson correlation coefficient (PCC) values were calculated using the JACoP v2.1.4 plugin for imageJ.[6]

8.1 Cytotoxicity Assays

Cytotoxicity of the S-APTRA-Rosamine was assessed using an automated assay. NIH 3T3 cells were grown in 24-well plates at 37°C and 5 % CO_2 to circa 90 % confluency in 500 µL media. The media was removed, and cells were dosed with 500 µL of the selected dye as a solution in media at the desired concentration. Incubation for the required time was followed by removal of the media containing the dye, rinsing with 400 µL phosphate buffer solution (PBS), and addition of 200 µL of 0.25 % Trypsin in PBS. After 3 minutes, 500 µL media was added to inhibit the activity of Trypsin, and the solution was agitated with the pipette to suspend the cells. The solution was placed in a cryovial, shaken, and a Via1-Cassette™ (ChemoMetec) withdrew 60 µL of cell suspension. The cassette was analysed using a NucleoCounter® NC-3000TM (ChemoMetec) cell counter to assess the cell viability as a percentage of live and dead cells. 3 replicates for each set of conditions were carried out.

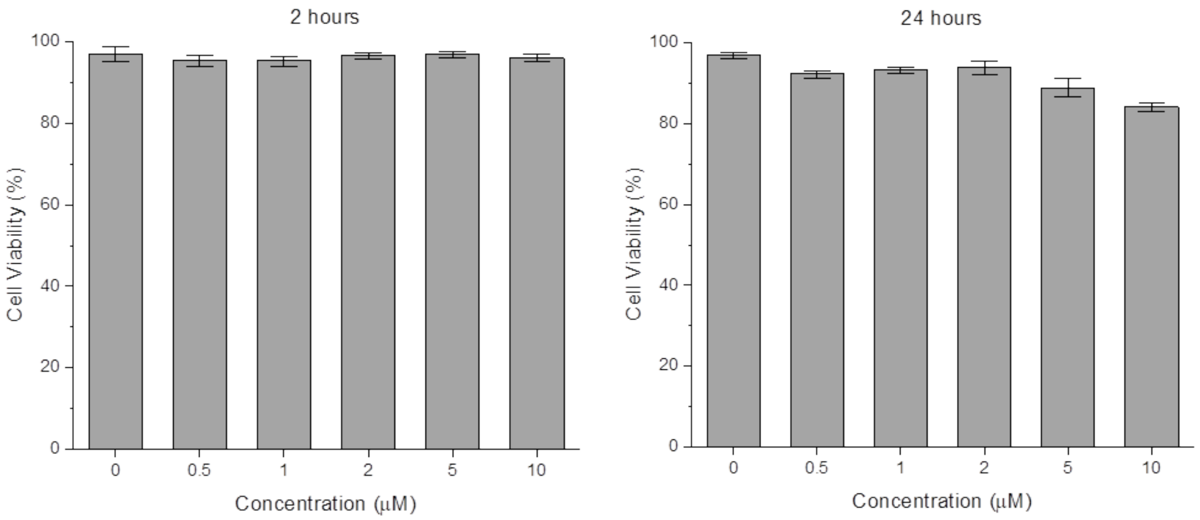

Figure S13. *Cytotoxicity data for S-APTRA-Rosamine after incubation at concentrations ranging from 0 to 10 µM at 2 hours, and 24 hours.*

8.2 Transmission Images

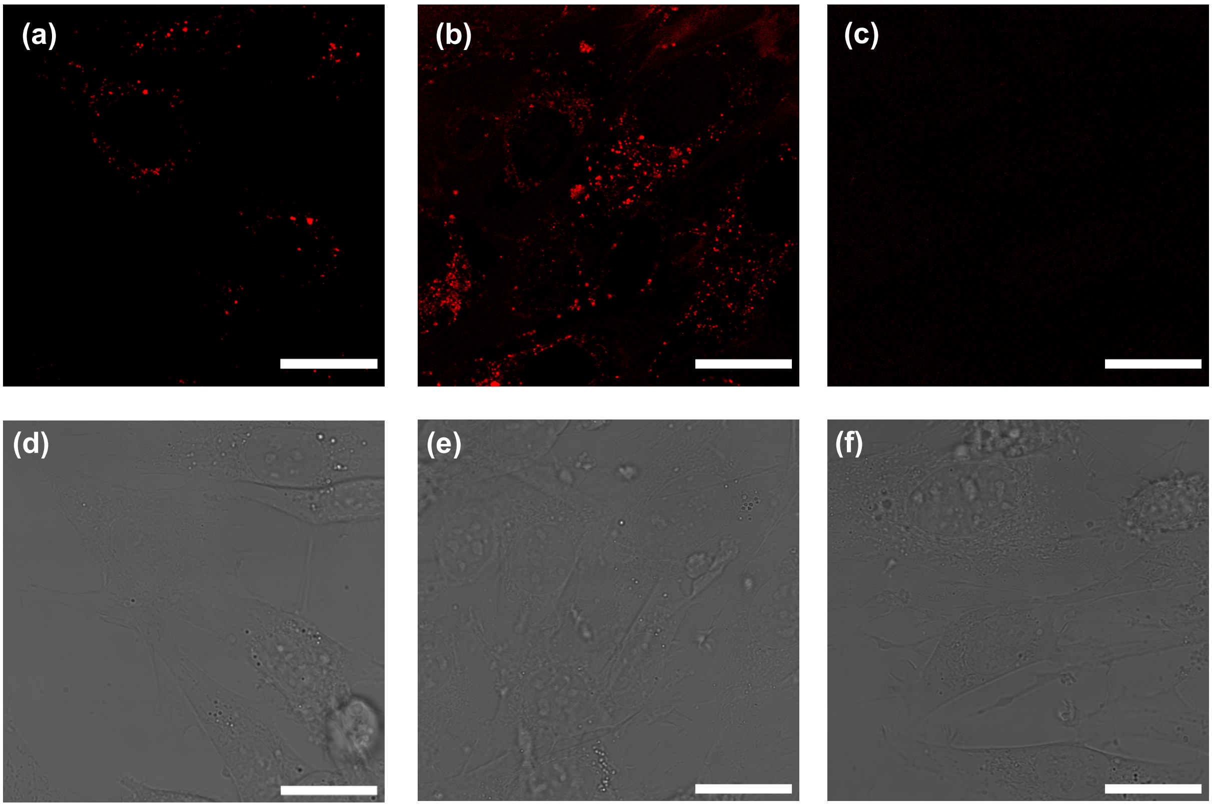

Figure S14. *Live cell fluorescence images from Figure 10 (a) – (c), shown here with the corresponding transmission images below (d) – (e).*

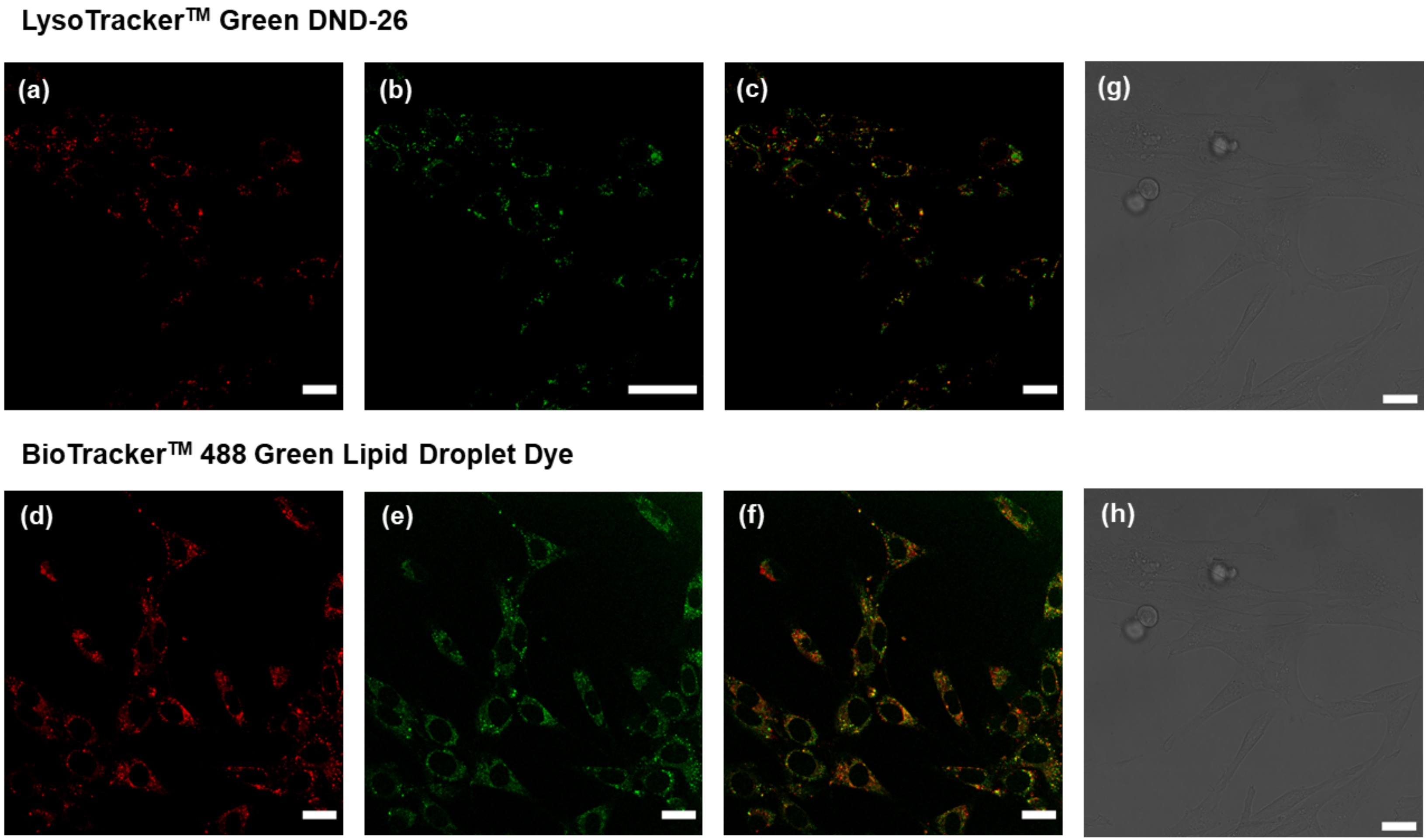

Figure S15. *Live cell fluorescence images from Figure 11 (a) – (c) and (d) – (f), shown here with the corresponding transmission images, (g) and (h) respectively.*

9. ^{1}H and $^{13}C\{^{1}H\}$ NMR Spectra

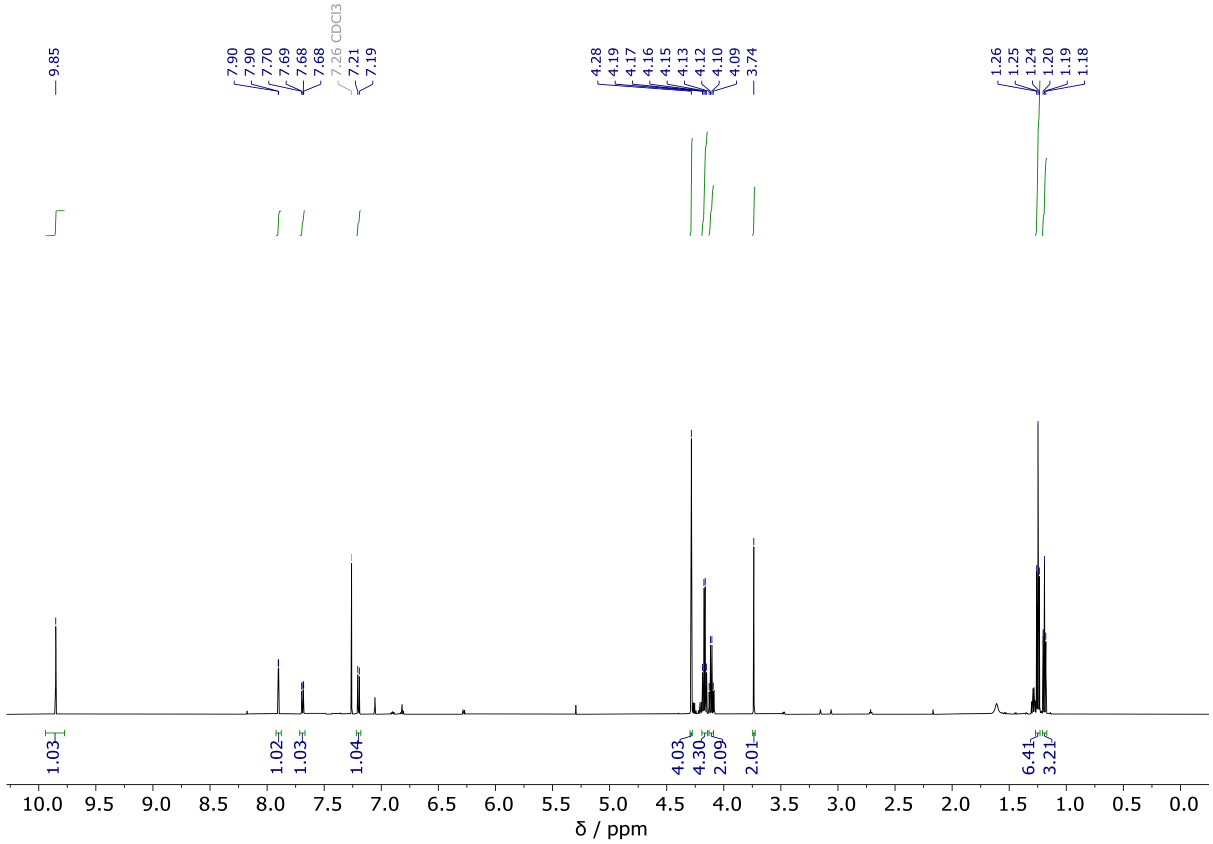

Figure S16. *^{1}H NMR spectrum of 4-formyl-S-APTRA-Et$_3$ in CDCl$_3$.*

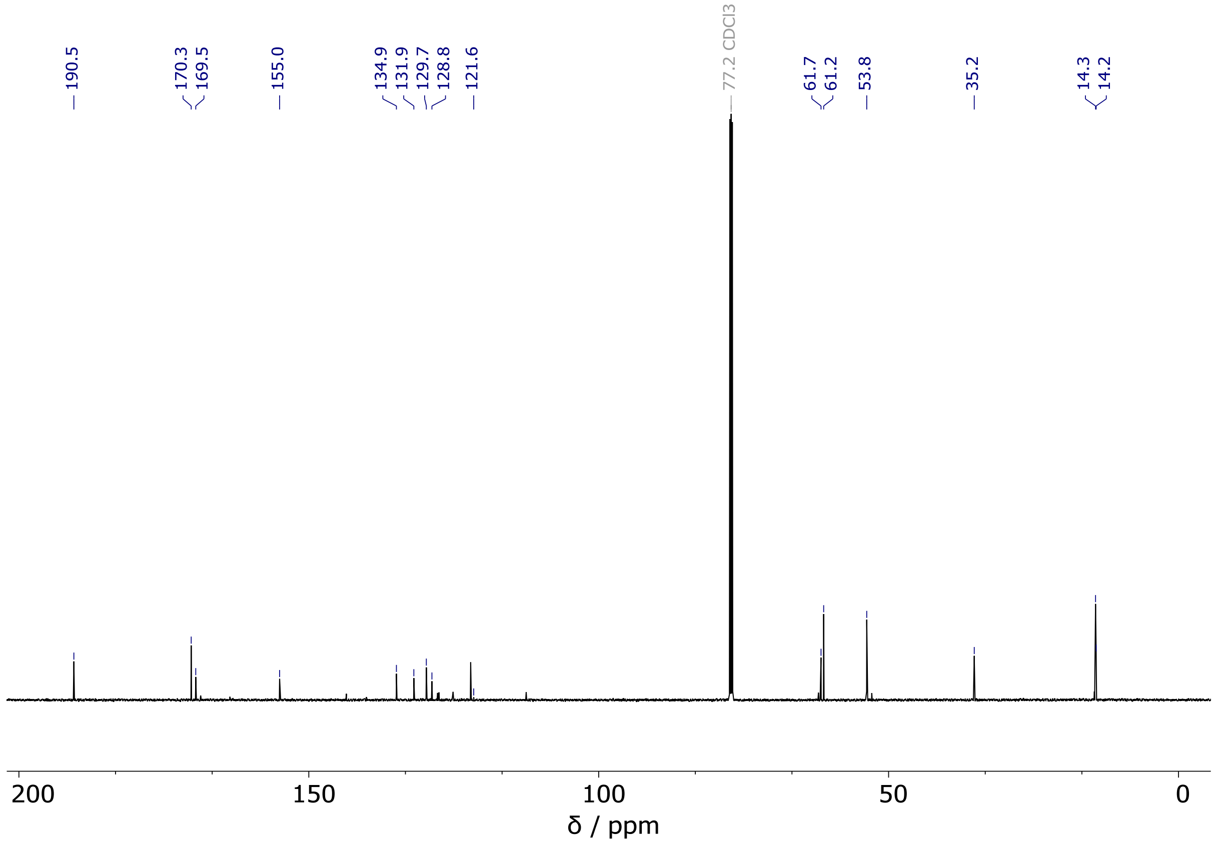

Figure S17. *$^{13}C\{^{1}H\}$ NMR spectrum of 4-formyl-S-APTRA-Et$_3$ in CDCl$_3$.*

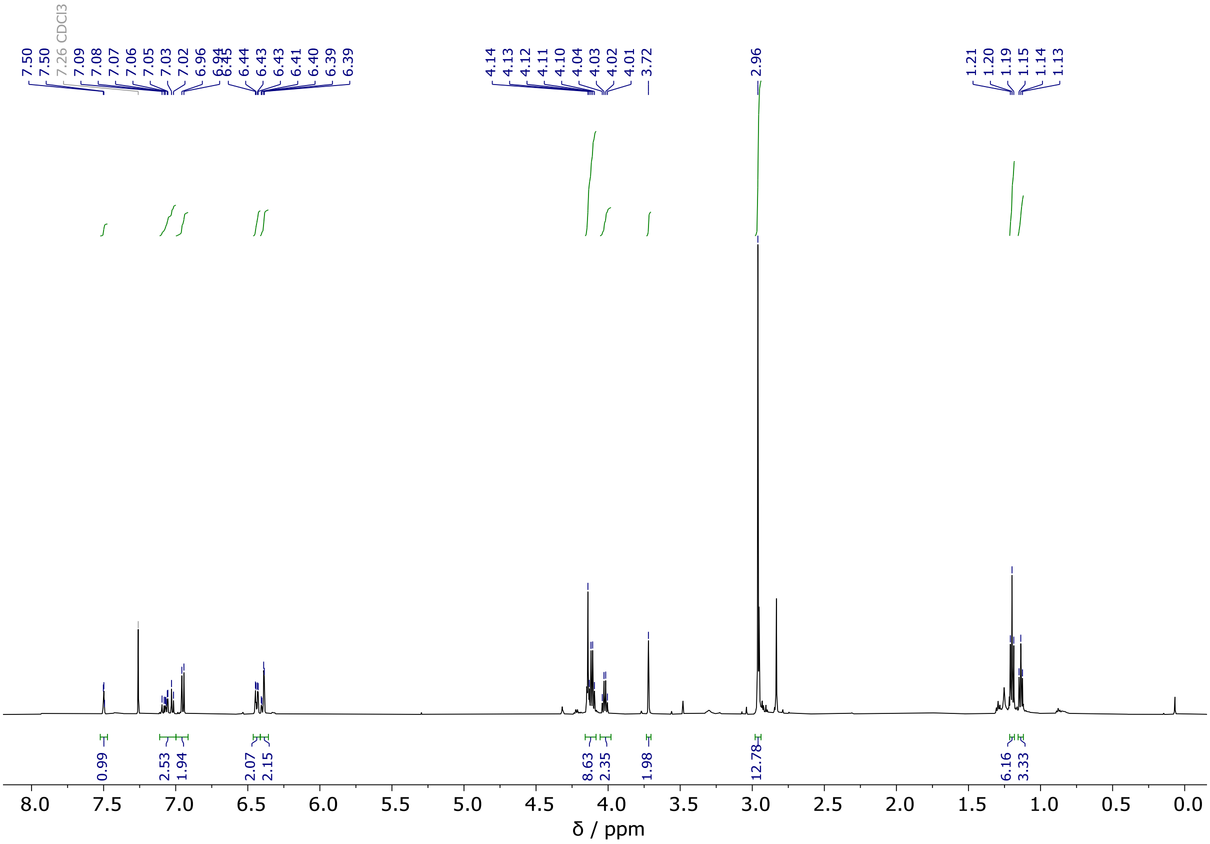

Figure S18. *^{1}H NMR spectrum of S-APTRA-Rosamine-Et$_3$ in CDCl$_3$.*

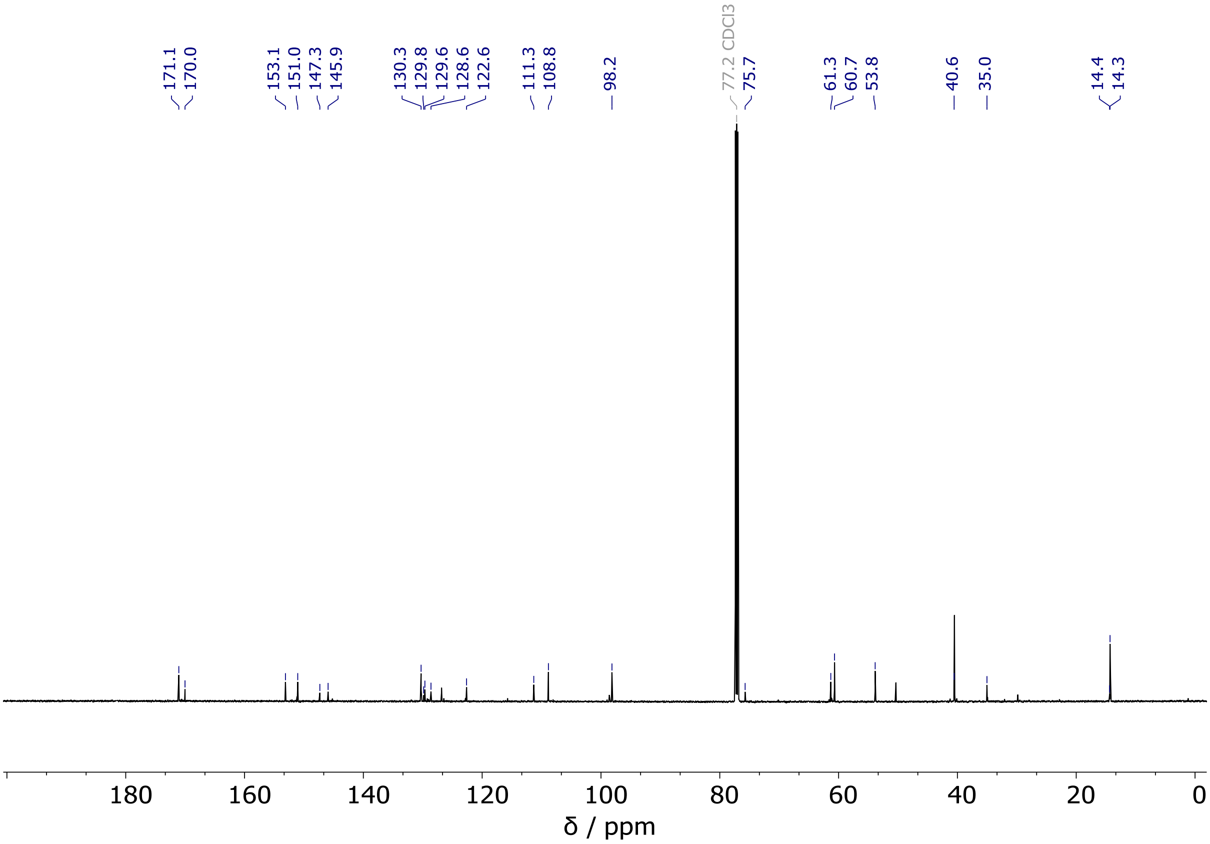

Figure S19. *^{13}C{^{1}H} NMR spectrum of S-APTRA-Rosamine-Et$_3$ in CDCl$_3$.*

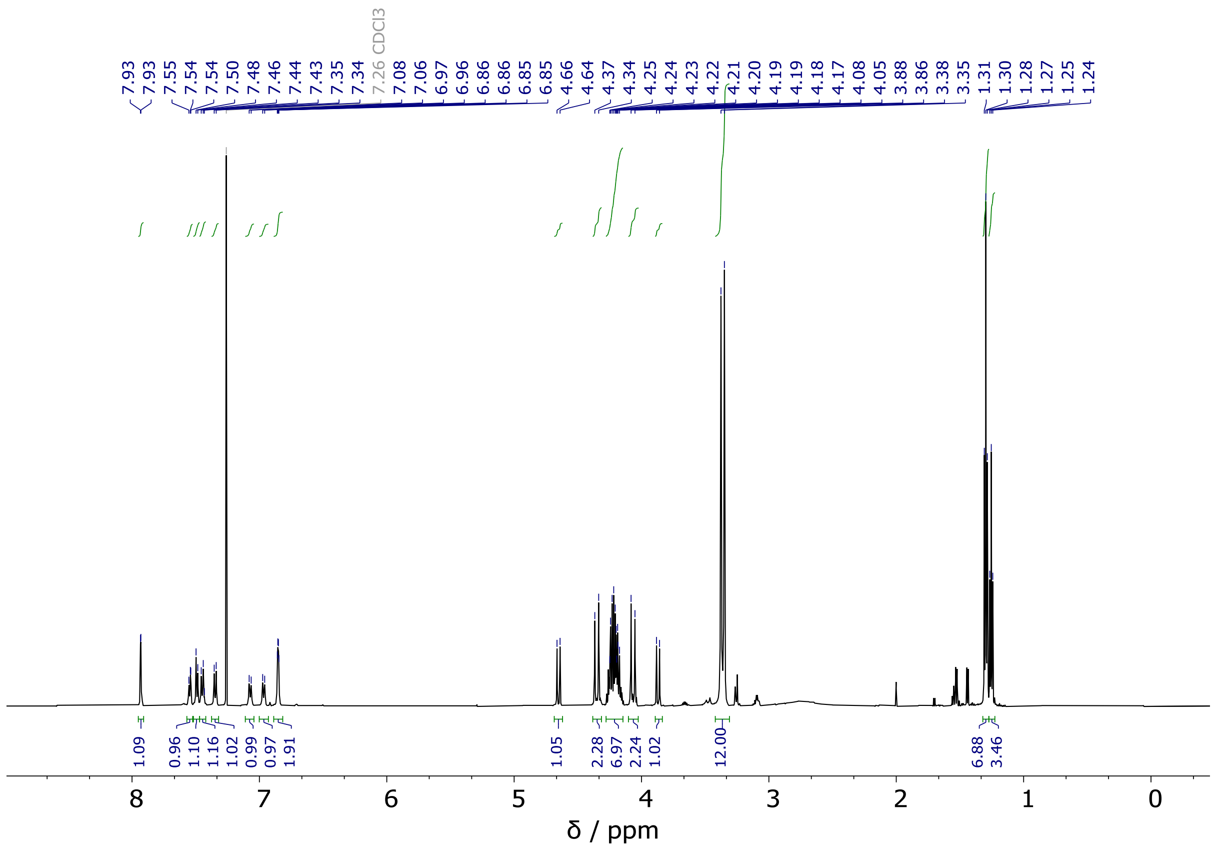

Figure S20. *^{1}H NMR spectrum of SO-APTRA-Rosamine-Et$_3$ in CDCl$_3$.*

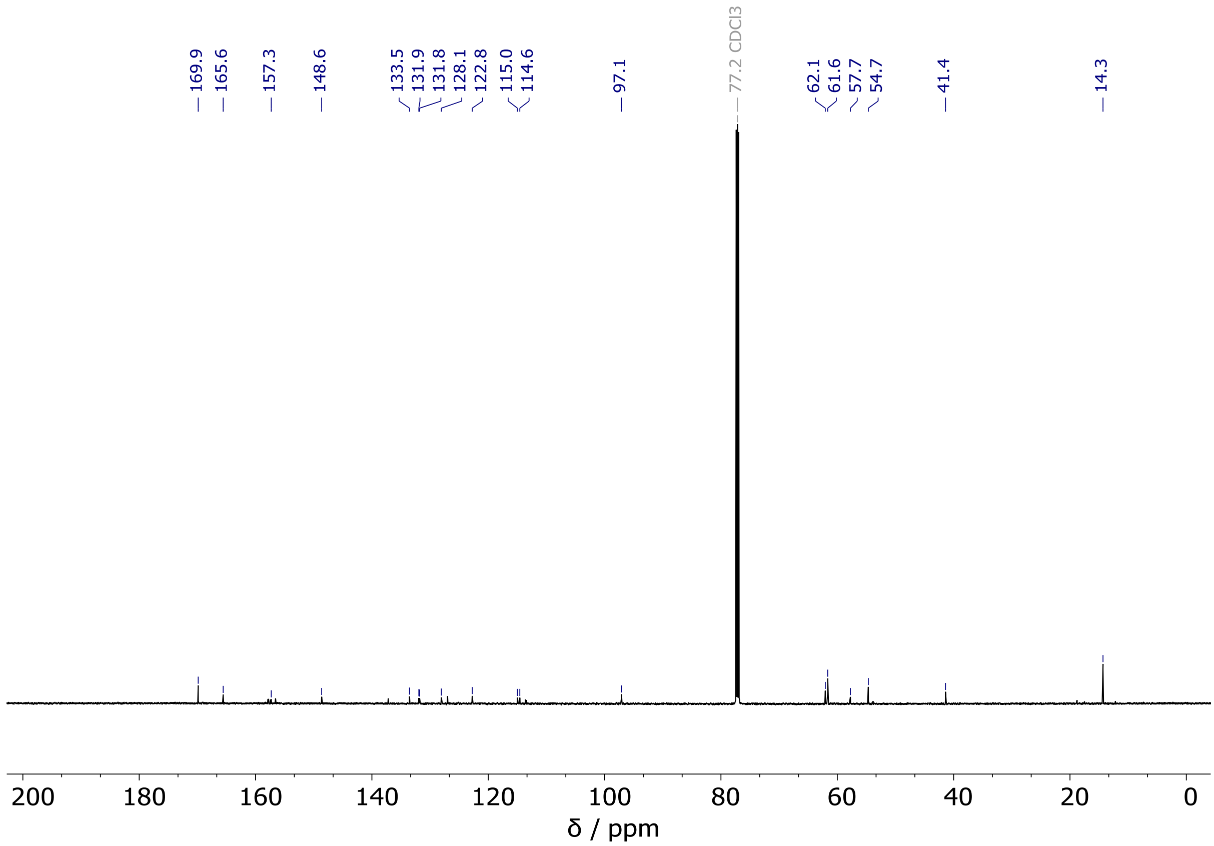

Figure S21. *^{13}C{^{1}H} NMR spectrum of SO-APTRA-Rosamine-Et$_3$ in CDCl$_3$.*

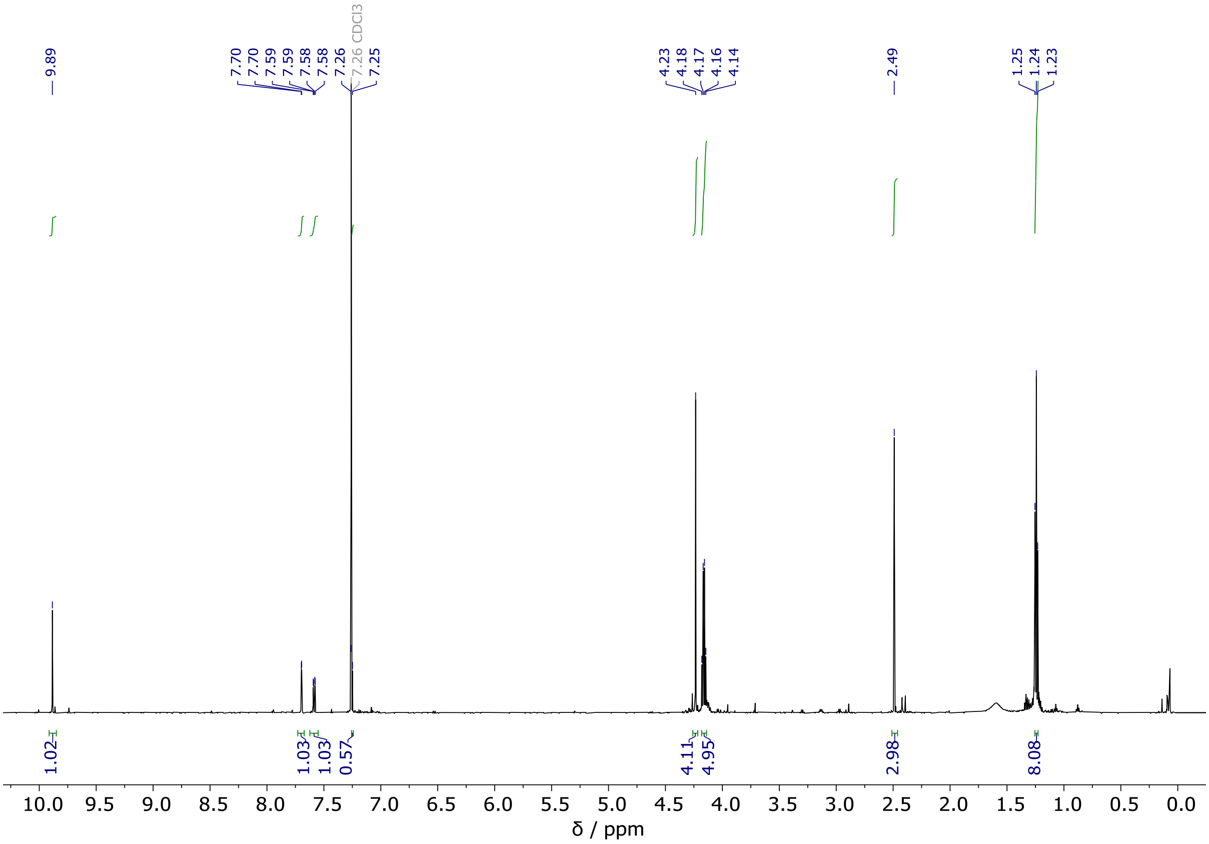

Figure S22. *^{1}H NMR spectrum of 4-formyl-S-APDIA-Et$_2$ in $CDCl_3$.*

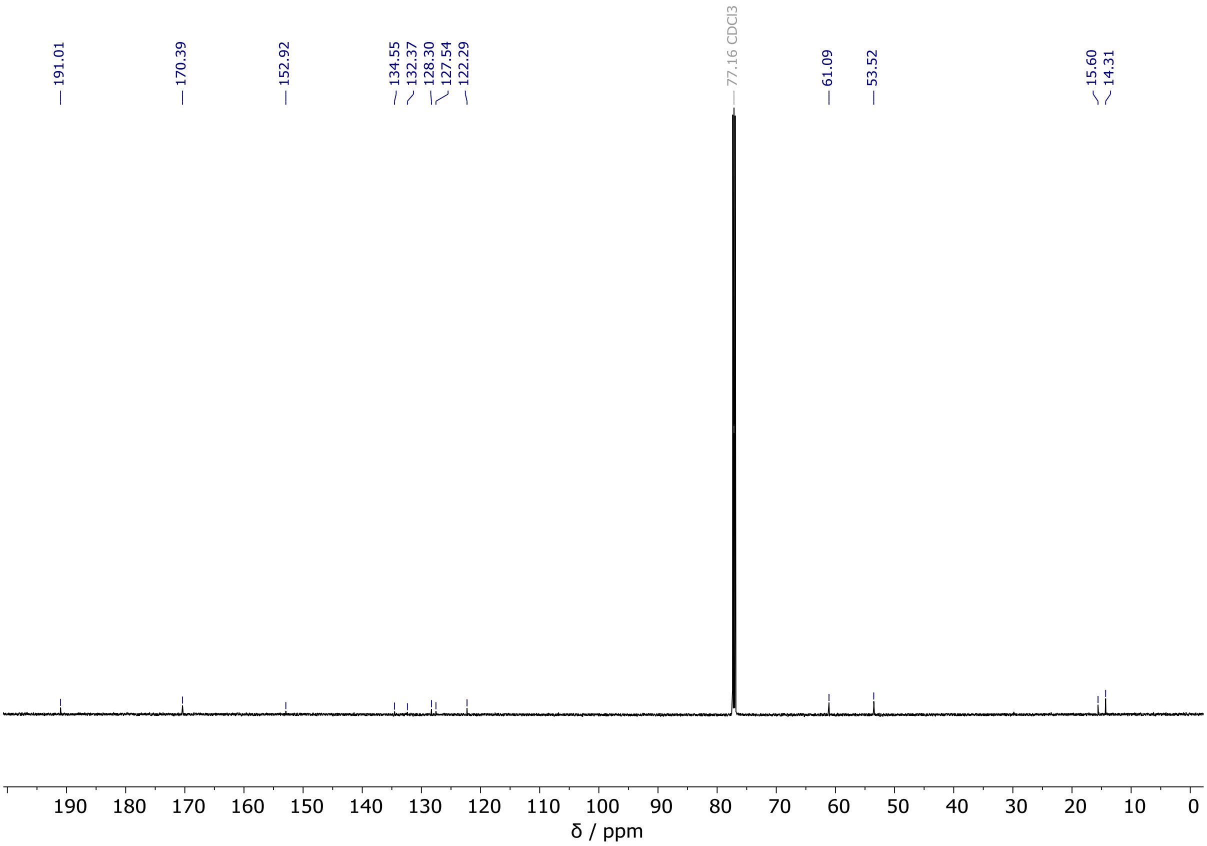

Figure S23. *$^{13}C\{^{1}H\}$ NMR spectrum of 4-formyl-S-APDIA-Et$_2$ in $CDCl_3$.*

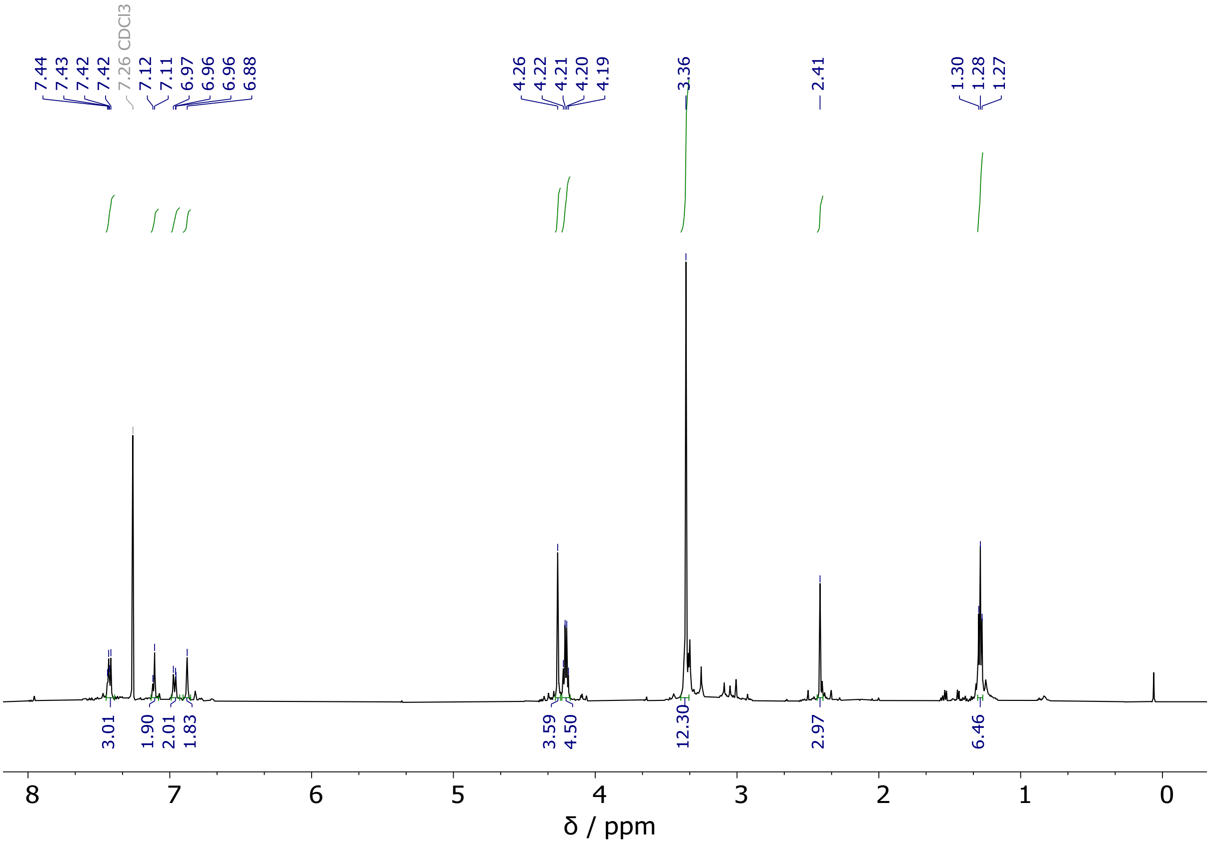

Figure S24. *^{1}H NMR spectrum of S-APDIA-Rosamine-Et$_2$ in $CDCl_3$.*

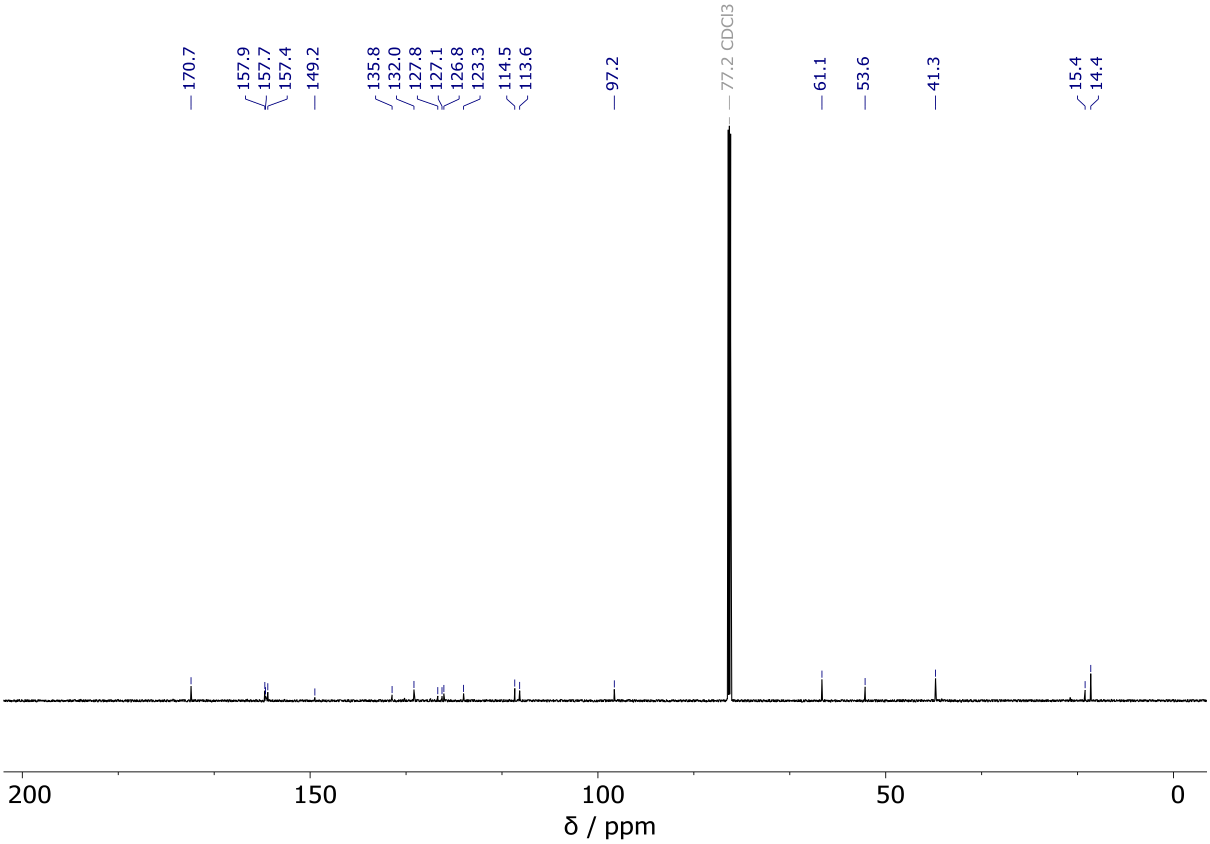

Figure S25. *$^{13}C\{^{1}H\}$ NMR spectrum of S-APDIA-Rosamine-Et$_2$ in $CDCl_3$.*

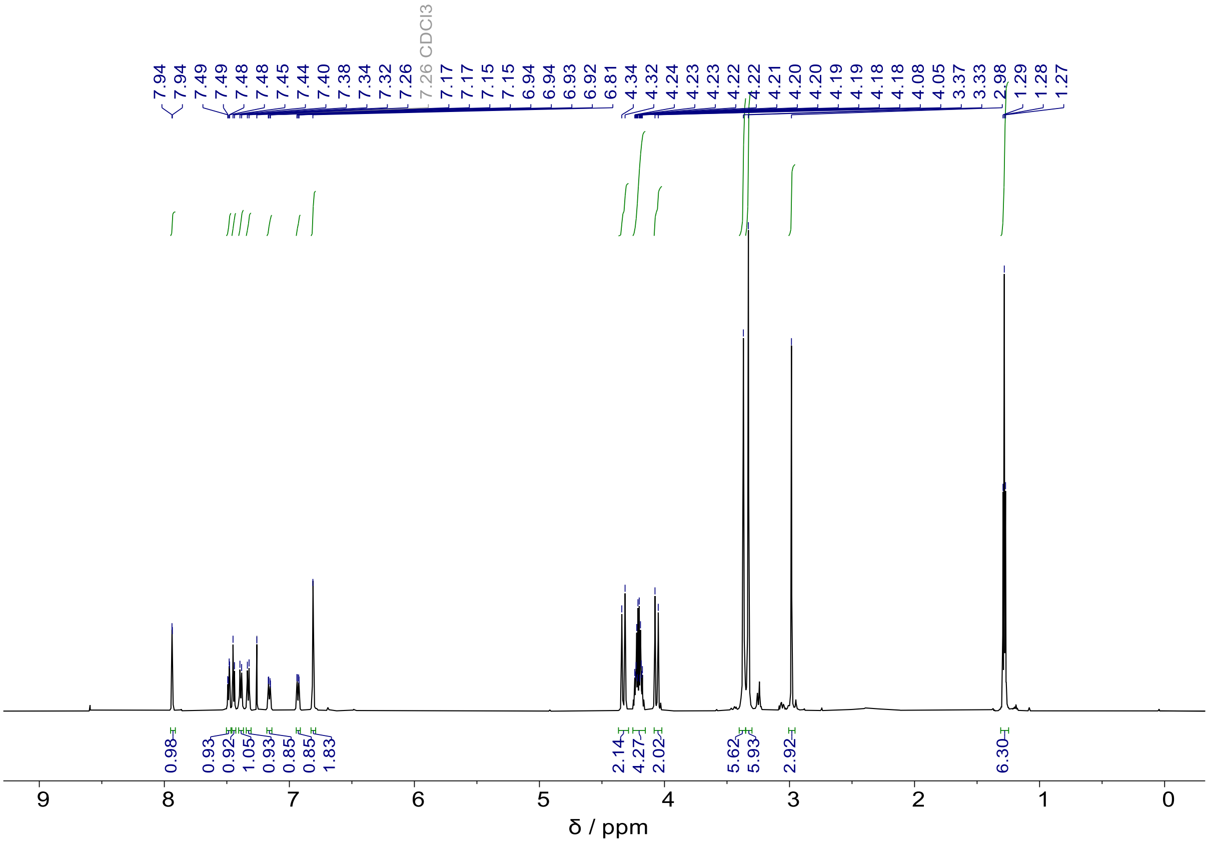

Figure S26. *^{1}H NMR spectrum of SO-APDIA-Rosamine-Et$_2$ in CDCl$_3$.*

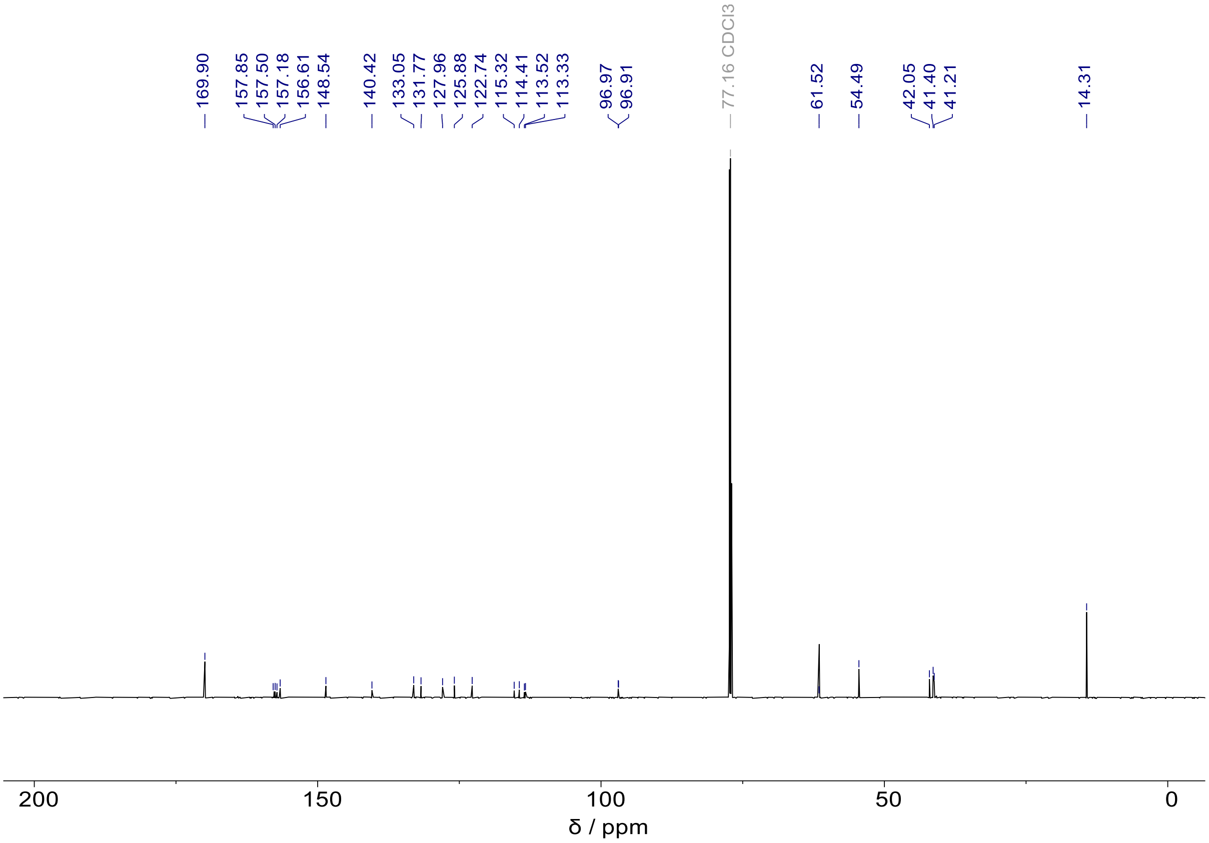

Figure S27. *^{13}C{^{1}H} NMR spectrum of SO-APDIA-Rosamine-Et$_2$ in CDCl$_3$.*

10. ESI-LRMS Spectra

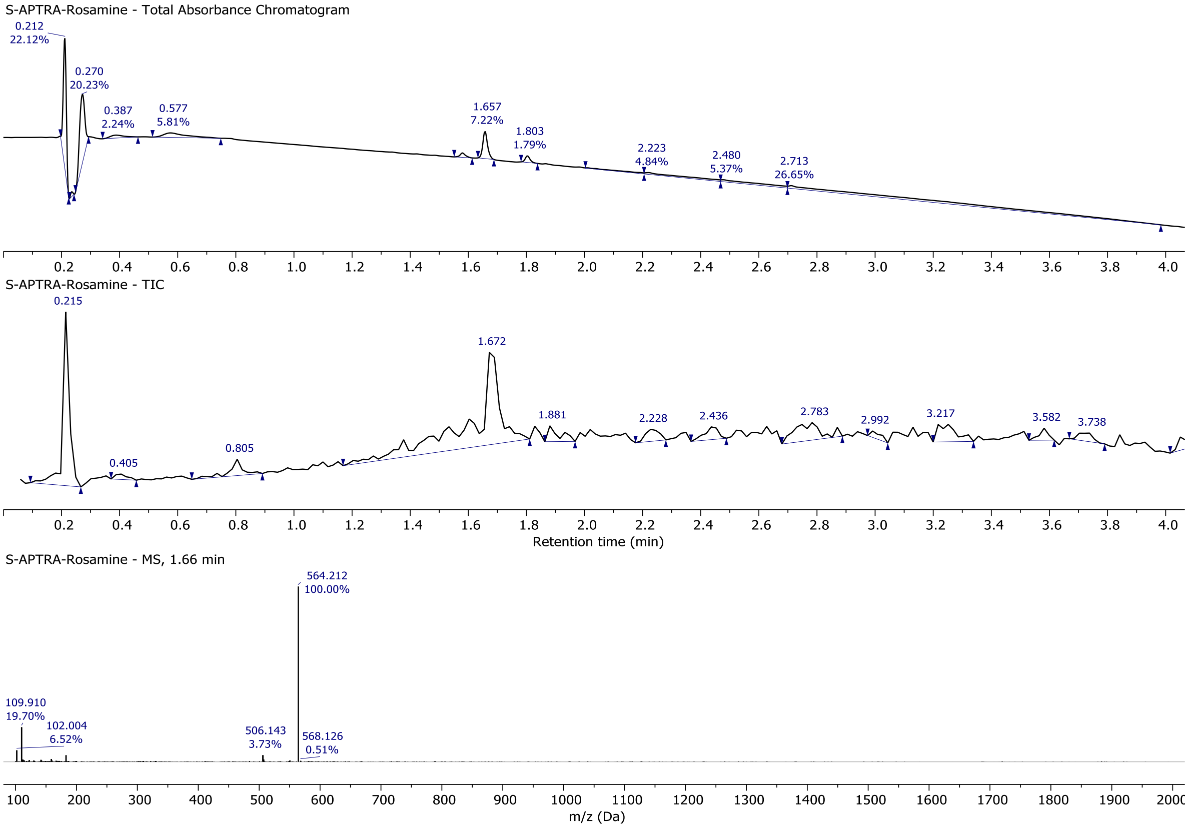

***Figure S28**. ESI-LRMS of S-APTRA-Rosamine.*

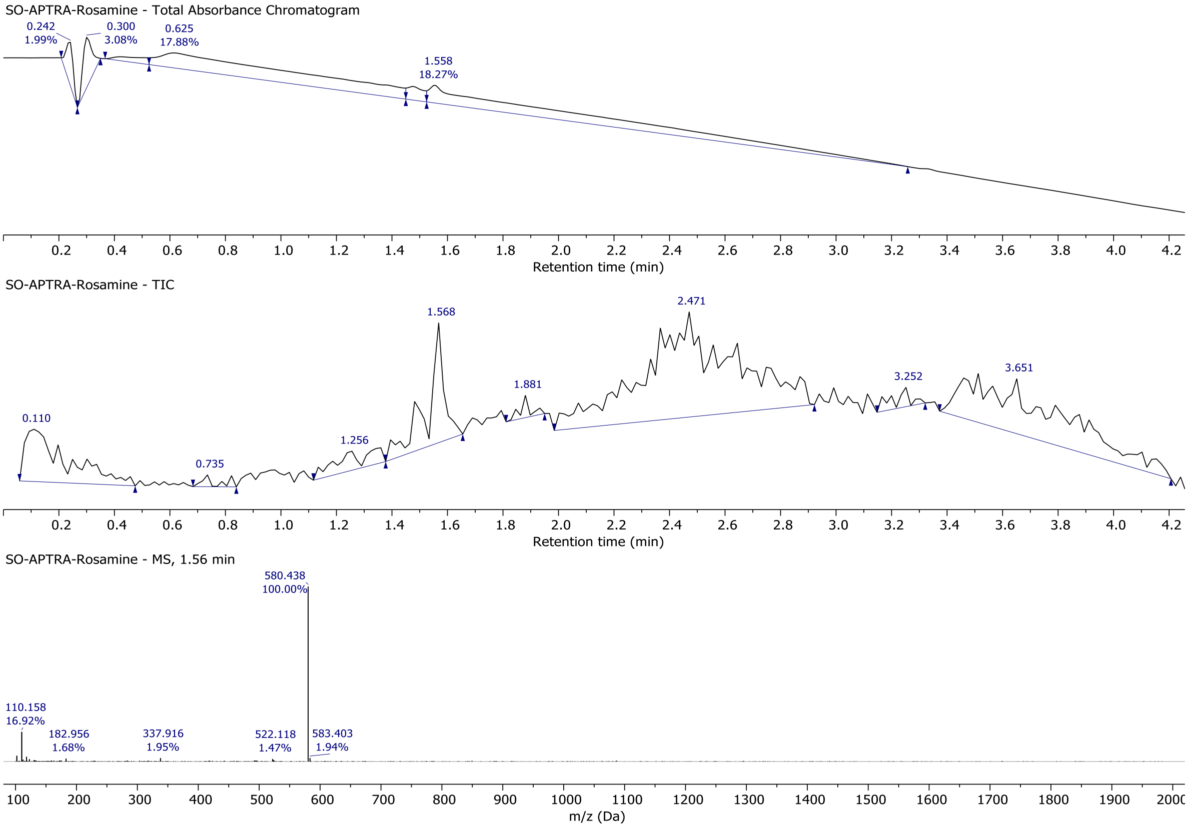

***Figure S29**. ESI-LRMS of SO-APTRA-Rosamine.*

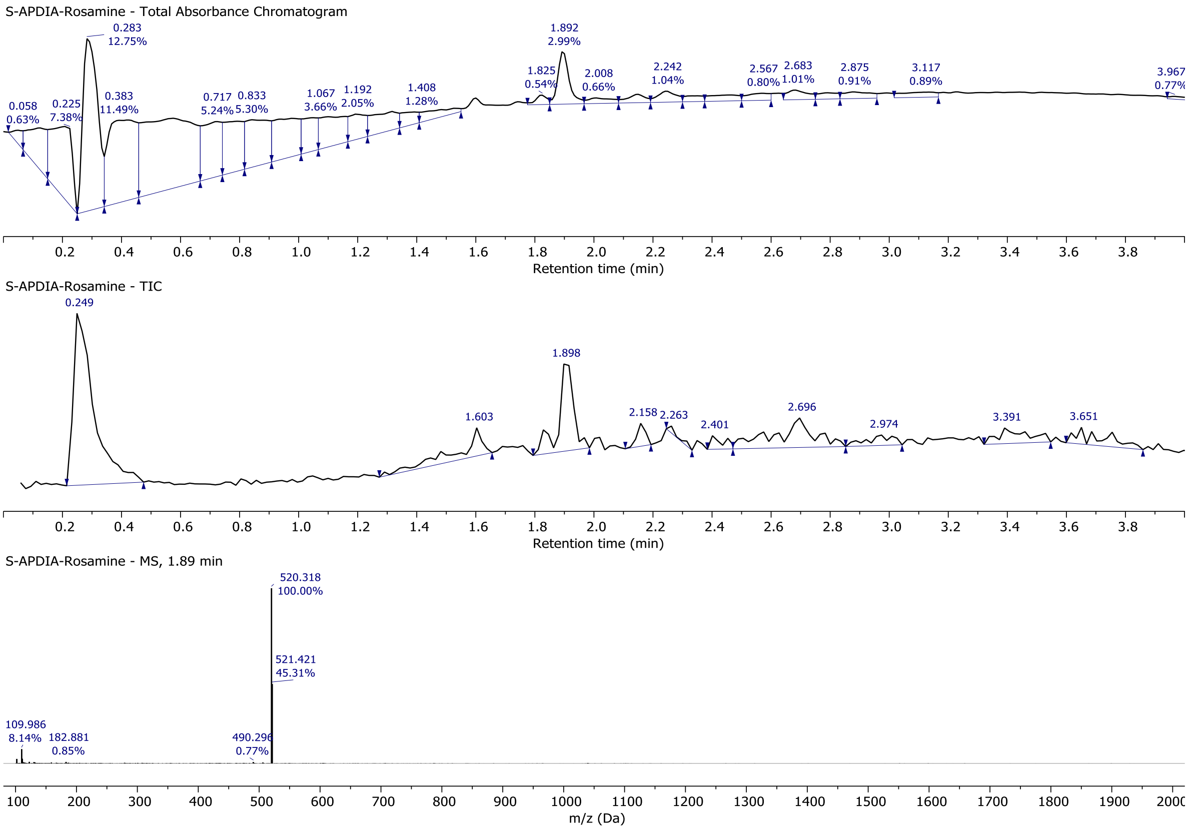

***Figure S30**. ESI-LRMS of S-APDIA-Rosamine.*

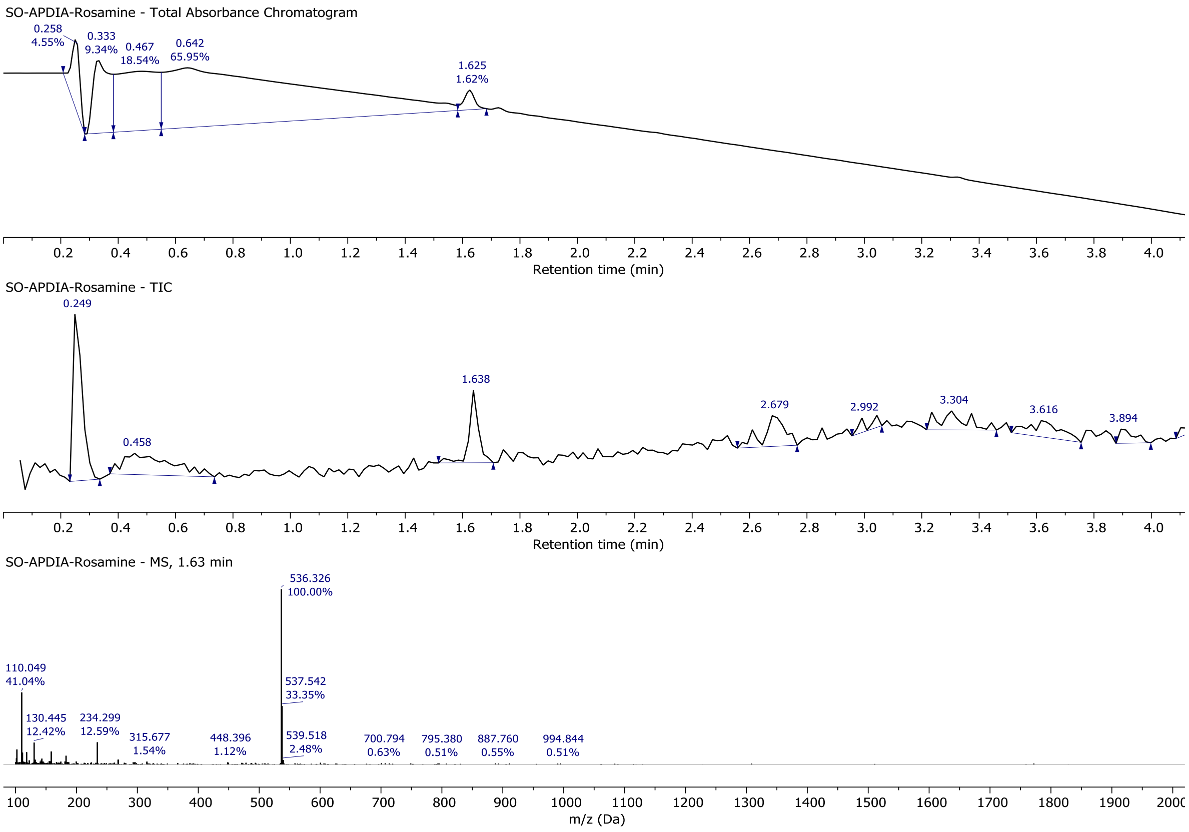

***Figure S31.** ESI-LRMS of SO-APDIA-Rosamine*

11. References

1. C. Hogg, L. L. Duncan, D. Parker and J. A. G. Williams, Enhanced binding of Zn^{2+} using a sulfur version of *o*-aminothiophenoltriacetate (APTRA): Introducing S-APTRA and derivatives. *Inorg. Chem.*, 2025, **64**, 9509–9518.
2. R. F. Kubin and A. N. Fletcher, Fluorescence quantum yields of some rhodamine dyes. *J. Lumin.* 1982, **27**, 455–462.
3. P. Thordarson, Determining association constants from titration experiments in supramolecular chemistry. *Chem. Soc. Rev.*, 2011, **40**, 1305–1323.
4. C. J. Fahrni and T. V. O'Halloran, Aqueous coordination chemistry of quinoline-based fluorescence probes for the biological chemistry of zinc. *J. Am. Chem. Soc.*, 1999, **121**, 11448–11458.
5. R. Pal, Phase modulation nanoscopy: a simple approach to enhanced optical resolution. *Faraday Discuss.*, 2015, **177**, 507–515.
6. S. Bolte and F. P. Cordelieres, A guided tour into subcellular colocalization analysis in light microscopy. *J. Microscopy*, 2006, **224**, 213–232.
